# The Autoimmune Disorder Susceptibility Gene *CLEC16A* Restrains NK Cell Function in YTS NK Cell Line and *Clec16a* Knockout Mice

**DOI:** 10.3389/fimmu.2019.00068

**Published:** 2019-02-01

**Authors:** Rahul Pandey, Marina Bakay, Heather S. Hain, Bryan Strenkowski, Anastasiya Yermakova, Jake A. Kushner, Jordan S. Orange, Hakon Hakonarson

**Affiliations:** ^1^The Center for Applied Genomics, The Children's Hospital of Philadelphia, Philadelphia, PA, United States; ^2^Section of Immunology, Allergy, and Rheumatology, Department of Pediatric Medicine, Texas Children's Hospital, Houston, TX, United States; ^3^Section of Pediatric Diabetes and Endocrinology, Department of Pediatric Medicine, Endocrine-Metabolism, Texas Children's Hospital, Houston, TX, United States; ^4^Department of Pediatrics, The Perelman School of Medicine, University of Pennsylvania, Philadelphia, PA, United States

**Keywords:** CLEC16A, NK cells, cytotoxicity, receptors, cell surface, autophagy

## Abstract

*CLEC16A* locus polymorphisms have been associated with several autoimmune diseases. We overexpressed *CLEC16A* in YTS natural killer (NK) cells and observed reduced NK cell cytotoxicity and IFN-γ release, delayed dendritic cell (DC) maturation, decreased conjugate formation, cell-surface receptor downregulation and increased autophagy. In contrast, siRNA mediated knockdown resulted in increased NK cell cytotoxicity, reversal of receptor expression and disrupted mitophagy. Subcellular localization studies demonstrated that CLEC16A is a cytosolic protein that associates with Vps16A, a subunit of class C Vps-HOPS complex, and modulates receptor expression via autophagy. *Clec16a* knockout (KO) in mice resulted in altered immune cell populations, increased splenic NK cell cytotoxicity, imbalance of dendritic cell subsets, altered receptor expression, upregulated cytokine and chemokine secretion. Taken together, our findings indicate that CLEC16A restrains secretory functions including cytokine release and cytotoxicity and that a delicate balance of CLEC16A is needed for NK cell function and homeostasis.

## Introduction

*CLEC16A* is a well-established autoimmune disorder susceptibility gene and has been associated with several autoimmune diseases, including type-1 diabetes (1–5), multiple sclerosis (6), primary adrenal insufficiency (7), Crohn's disease (8), primary biliary cirrhosis (9), juvenile idiopathic arthritis (10), rheumatoid arthritis (10), and alopecia areata (11), suggesting that *CLEC16A* could be a master regulator of aberrant autoimmune responses. Despite the strong association of *CLEC16A* across numerous autoimmune and inflammatory disorders, little is known about CLEC16A's physiological function or its role in disease pathogenesis. Several studies have described the role of CLEC16A in autophagy processes (12–14). Previous studies show that loss of CLEC16A leads to an Nrdp1 targeting of Parkin, a master regulator of mitophagy (15), and that golgi-associated CLEC16A negatively regulates autophagy via modulation of mTOR activity (16). How this relates to the autoimmune function is yet to be determined.

NK cells are critical facilitators of innate immune responses and host defense. They are efficient producers of proinflammatory cytokines and mediate cytotoxic activity that could directly trigger autoimmunity through killing host cells or indirectly by interacting with antigen-presenting cells (APC) or with T cells (17). Both a disease-controlling and a disease-promoting role have been suggested for NK cells in human autoimmune conditions. Through their potential autoreactivity or interactions with other cells, including dendritic cells (DCs), macrophages or T lymphocytes, they can induce excessive inflammation or favor adaptive autoimmune responses (18). Thus, NK cells are in a prime position to militate the onset, maintenance and progression of autoimmune diseases under different circumstances.

In our previous work in type-1 diabetes (2), the protective *CLEC16A* alleles were associated with higher levels of CLEC16A (formally known as *KIAA0350*). We also recently showed that ubiquitous loss of CLEC16A led to disrupted mitophagy in immune cells (19). In view of the above, we hypothesized that *CLEC16A* functions in NK cells to restrain secretory functions including cytokine release and cytotoxicity.

In this study, we designed experiments to better define the role of CLEC16A in NK cells, inflammation, and autoimmune disorders. We show that CLEC16A is a cytosolic protein that exhibits differential expression patterns in human immune cells, including NK cells. CLEC16A also interacts with the class C Vps-HOPS complex to modulate cell surface receptor expression. We also show that siRNA mediated knockdown results in increased NK cell cytotoxicity, reversal of receptor expression, and disrupted mitophagy, whereas, overexpression leads to reduced NK cell killing, IFN-γ release and DC maturation. Importantly, we found that overexpression of CLEC16A promotes autophagy while knockdown/knockout triggers disrupted mitophagy. When addressing the role of *Clec16a* in knockout mice, we observed altered splenic immune cell population, increased splenic NK cell cytotoxicity, up-regulated cytokine and chemokine secretion, imbalance in dendritic cell subsets, altered receptor expression and inflammatory phenotype, all of which support a key role of CLEC16A in autoimmunity.

## Results

### CLEC16A Expression in Human Immune Cells, Including NK Cell Lines

**Figure 1:**
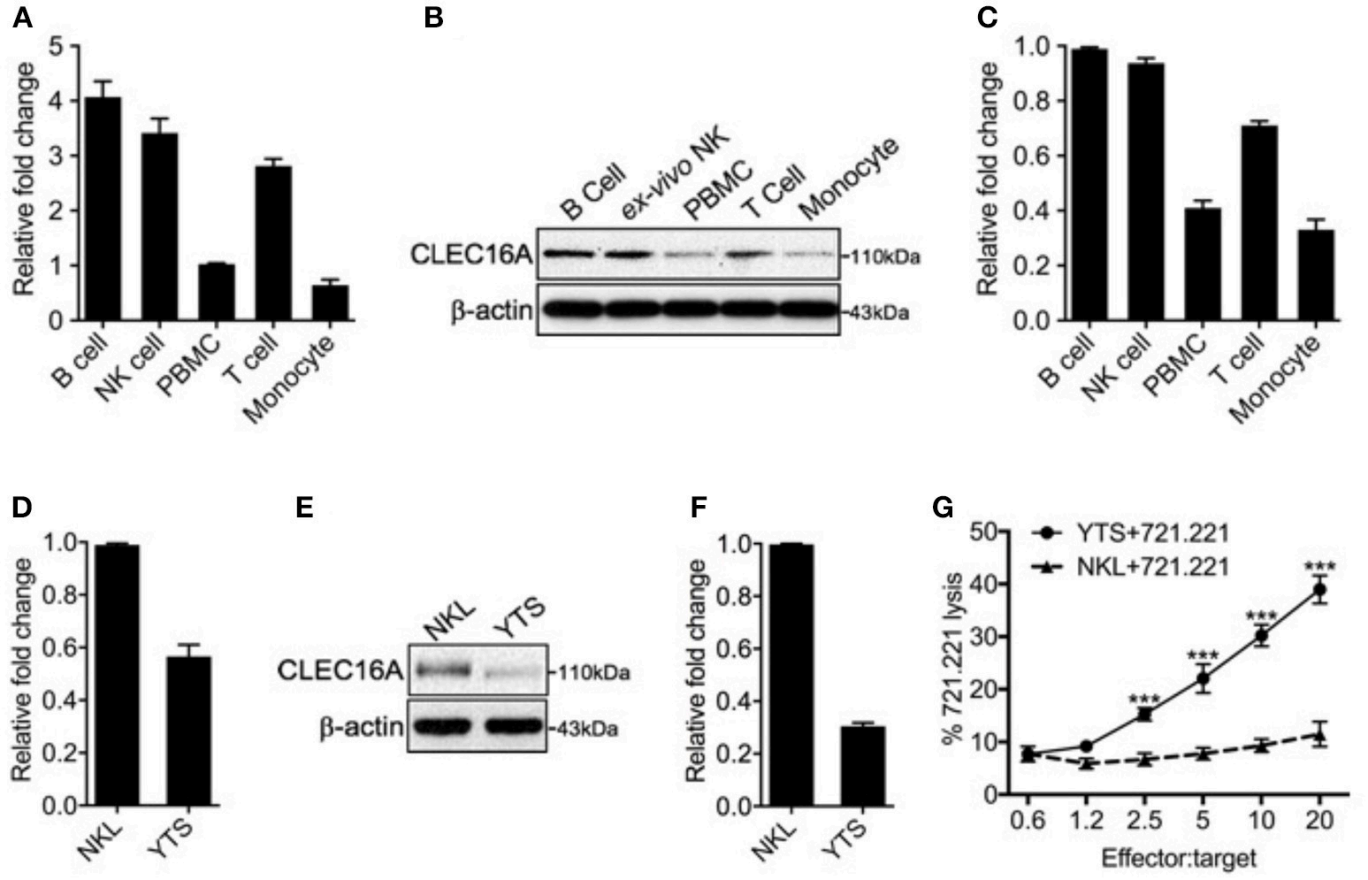
Differential *CLEC16A* expression in human immune cells and NK cell lines. **(A)** Relative *CLEC16A* mRNA expression in human B, NK, PBMC, T-cells, and monocyte by RT-PCR (*n* = 3 repeats). **(B)** Representative Western blot analysis from human B, NK, PBMC, T-cells, and monocytes for CLEC16A expression. **(C)** Quantitation graph depicting CLEC16A protein expression (*n* = 3 repeats). **(D)** Relative *CLEC16A* mRNA expression by RT-PCR in two immortalized human NK cell lines (NKL and YTS). **(E)** Representative Western blot depicting CLEC16A expression in NKL and immortalized NK cell lines. **(F)** Quantitation graph depicting CLEC16A protein expression (*n* = 3 repeats). **(G)** Cytotoxicity graph depicting killing of ^51^Cr-labeled 721.221 using YTS and NKL as effectors. Data represents means ± SE of five independent experiments (*n* = 5). *CLEC16A* mRNA expression was normalized to β-actin. The top panel of immunoblot shows CLEC16A expression. Membranes were stripped and re-probed for β-actin as a loading control (bottom panel). ^***^P < 0.001 (unpaired two-tailed Student's *t*-test).

We assessed the expression of *CLEC16A* at mRNA and protein levels in human immune cells and two NK cell lines, using TaqMan probes and immunoblot analysis. In the immune cell types investigated, *CLEC16A* was highly expressed in B, NK, and T cells at the mRNA level (Figure 1A). CLEC16A protein was detected in all immune cell types examined, with the highest protein expression found in B cells followed by NK and T cells (Figure 1B). Importantly, protein expression correlated with mRNA expression levels (Figures 1A,C). In our evaluation of CLEC16A expression in two NK cell lines that were homozygous for either the protective [A/A] allele (NKL) or non-protective [G/G] (YTS) alleles of rs2903692, CLEC16A expression was higher in the NKL cell line at both mRNA and protein levels (Figures 1D–F). We predicted that possessing the protective allele [A/A] would result in restrained NK cell functions. We tested these two cell lines in a cytotoxicity assay and confirmed that the [A/A] allele results in restrained NK cell cytotoxicity in the NKL cell line (Figure 1G). In contrast, YTS possessing the non-protective allele showed significantly higher killing of 721.221 targets in comparison to NKL.

### CLEC16A Overexpression Constrains Cytotoxicity, IFN-γ Production, and DC-Maturation

**Figure 2:**
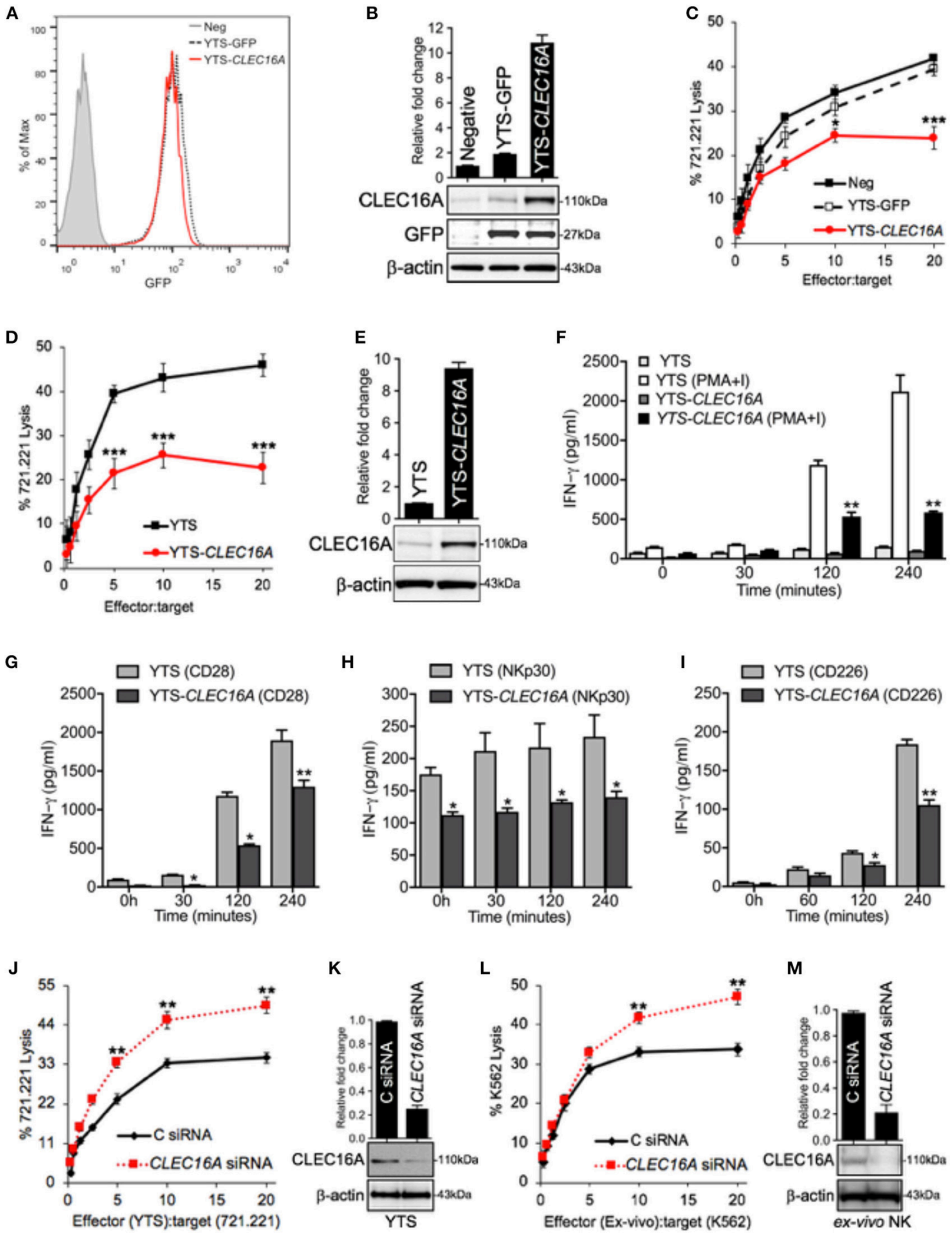
*CLEC16A* overexpression impairs cytotoxicity, IFN-γ production and DC maturation, whereas siRNA mediated knockdown enhances the cytotoxicity in NK cells. **(A)** Transduced YTSeco cells were sorted via FACS into population with comparable GFP expression and cultures had consistent GFP expression over time. **(B)** Negative, YTS-GFP, and YTS-*CLEC16A* cells analyzed for CLEC16A and GFP expression in an immunoblot analysis. Membranes were stripped and re-probed for β-actin as a loading control (bottom panel). Quantitation graph depicting CLEC16A expression (top panel; *n* = 3 repeats). **(C)** Cytotoxicity of negative control (mock transduced YTS cells), YTS-GFP and YTS-*CLEC16A* NK cell lines against ^51^Cr-labeled 721.221 targets. Data represents means ± SE of five independent experiments. **(D)** Cytotoxicity of parental YTS and YTS-*CLEC16A* NK cells against ^51^Cr-labeled 721.221 target. Data represents means±SE of three independent experiments **(E)** Representative Western blot of YTS and YTS-*CLEC16A* cells for CLEC16A expression and β-actin (bottom panel). Quantitation graph depicting CLEC16A expression (top panel; *n* = 3 repeats). **(F,I)** CLEC16A overexpression limits IFN-γ production in YTS-*CLEC16A*. IFN-γ release measured by ELISA in YTS and YTS-*CLEC16A* NK cell lines at the indicated time points. Data represents means ± SE of three independent experiments. **(F)** Cell lines were cultured alone or with PMA and ionomycin (PMA+I). **(G)** YTS and YTS-*CLEC16A* cells were activated with plate bound anti-CD28 for the indicated time points shown. **(H)** YTS and YTS-*CLEC16A* cells activated with plate bound anti-NKp30 for the indicated time points shown. **(I)** YTS and YTS-*CLEC16A* cells activated with plate bound anti-CD226 for the indicated time points and IFN-γ release shown. **(J)** siRNA knockdown of *CLEC16A* in parental YTS cells. 721.221 target cell killing by YTS cells, 24 h post nucleofection of the YTS cells with either *CLEC16A* or Control siRNAs. Data represents means ± SE of three independent experiments. **(K)** Representative Western blot depicting CLEC16A expression after siRNA mediated knockdown in YTS cells. Quantitation graph depicting CLEC16A expression (top panel; *n* = 3 repeats). **(l)** siRNA mediated knockdown of *CLEC16A* in *ex vivo* NK cells and cytotoxicity. K562 target cell killing by *ex vivo* NK cells, 24 h after nucleofection of the *ex vivo* NK cells with either *CLEC16A* or Control siRNAs. Data represents means ± SE of three independent experiments. **(M)** Representative Western blot depicting CLEC16A protein expression after knockdown in *ex vivo* NK cells. Quantitation graphdepicting CLEC16A expression (top panel; *n* = 3 repeats). ^*^P < 0.05, ^**^P < 0.01, ^***^P < 0.001 (unpaired two-tailed Student's *t*-test).

To investigate the functional importance of CLEC16A in NK cells, we generated a NK cell line stably overexpressing *CLEC16A*. Transduced YTS cells were identified through GFP expression and sorted by FACS to create a stable NK cell line overexpressing *CLEC16A* (Figure 2A). CLEC16A overexpression was validated by Western blot with GFP expression serving as an internal control (Figure 2B). To determine the impact of *CLEC16A* overexpression on NK cell function, we first evaluated cytotoxicity against 721.221 targets using a negative control (Neg), YTS-GFP and YTS-*CLEC16A* NK cell lines. Cytotoxicity of *CLEC16A* overexpressing cell lines revealed a significant decrease in killing of targets in comparison to negative control (Figure 2C). We also compared the parental YTS and YTS-*CLEC16A* NK cells and, as anticipated, we observed a significant decrease in cytotoxicity of YTS-*CLEC16A* cells (Figure 2D). CLEC16A overexpression was validated by Western blot (Figure 2E). These results provide the first line of evidence that CLEC16A serves a role in restraining the cytolytic function of NK cells.

The second major function of NK cells is the production of a wide variety of cytokines, chemokines and other regulators. NK cells are most appreciated for their ability to produce the IFN-γ, which has both anti-viral and immune enhancing capabilities (20). We evaluated if IFN-γ production was impaired by *CLEC16A* overexpression. Stimulation with PMA-Ionomycin of YTS-*CLEC16A* NK overexpressing cells resulted in a significant decrease in IFN-γ production at 120 and 240 min in comparison to YTS at both time points (Figure 2F). With receptor mediated anti-CD28 activation, the overexpressing NK cell line showed a significant decrease at 30, 120 and 240 min in comparison to the parental YTS cells (Figure 2G). With another form of receptor mediated activation (NKp30), the YTS-*CLEC16A* NK cells showed a significant decrease at all indicated time points in comparison to the parental YTS cells (Figure 2H). We also analyzed the effect of CD226 crosslinking on IFN-γ secretion. The YTS-*CLEC16A* NK cell line showed a significant decrease at 120 and 240 min in comparison to the parental YTS cells (Figure 2I). These observations are consistent with a role of CLEC16A in restraining cytokine release.

To determine if CLEC16A overexpression has any effect on NK-dependent DC maturation, DCs were generated using peripheral blood mononuclear cells (PBMCs) derived from healthy donors. To induce DC maturation, immature DCs were co-cultured with YTS or YTS-*CLEC16A* NK cells; medium with and without LPS served as negative and positive controls, respectively. DCs cultured for 2 days with NK cells acquired a cell surface phenotype typical of mature DCs. The expression of CD83 and CD86 were upregulated in comparison to the negative control (medium alone). In comparison to YTS NK cells, co-culture with YTS-*CLEC16A* NK cells showed a significant decrease in DC maturation, as reflected by a decrease in CD83 and CD86 expression (Supplementary Figure S1A). The mechanism by which YTS-*CLEC16A* fails to induce DC maturation could be attributed to decreased cell-to-cell contact and release of soluble factors. These results indicate that overexpression of CLEC16A in NK cells delays NK-dependent DC maturation. Thus, the NK/DC interaction should not just be considered as a cross-talk mechanism between two cell populations of innate cells but rather as a more complex cooperative network of cell subsets with actions in discrete regions of the body to fulfill complementary tasks.

To further confirm that CLEC16A acts to restrain NK cytolytic activity we used small-interfering (si)RNA to mediate knockdown in NK cells. 721.221-target cell killing by YTS NK cells was evaluated by ^51^Cr-release assay 24 h post nucleofection with either control or *CLEC16A* siRNA. As anticipated, reducing the protein expression resulted in a significant increase of cytotoxicity for YTS (Figure 2J). We confirmed these findings in *ex vivo* NK cells. K562 target cell killing using *ex vivo* NK cells were evaluated in ^51^Cr-release assay 24 h post nucleofection. Reducing the CLEC16A protein expression resulted in significant increase of cytotoxicity for *ex vivo* NK cells (Figure 2L). CLEC16A protein knockdown was validated by Western blot (Figures 2K,M). Collectively these results demonstrate that under normal conditions, CLEC16A plays a critical role in constraining major functions of NK cells.

### Overexpression Restrains Conjugate Formation and Migration in YTS-*CLEC16A*

**Figure 3:**
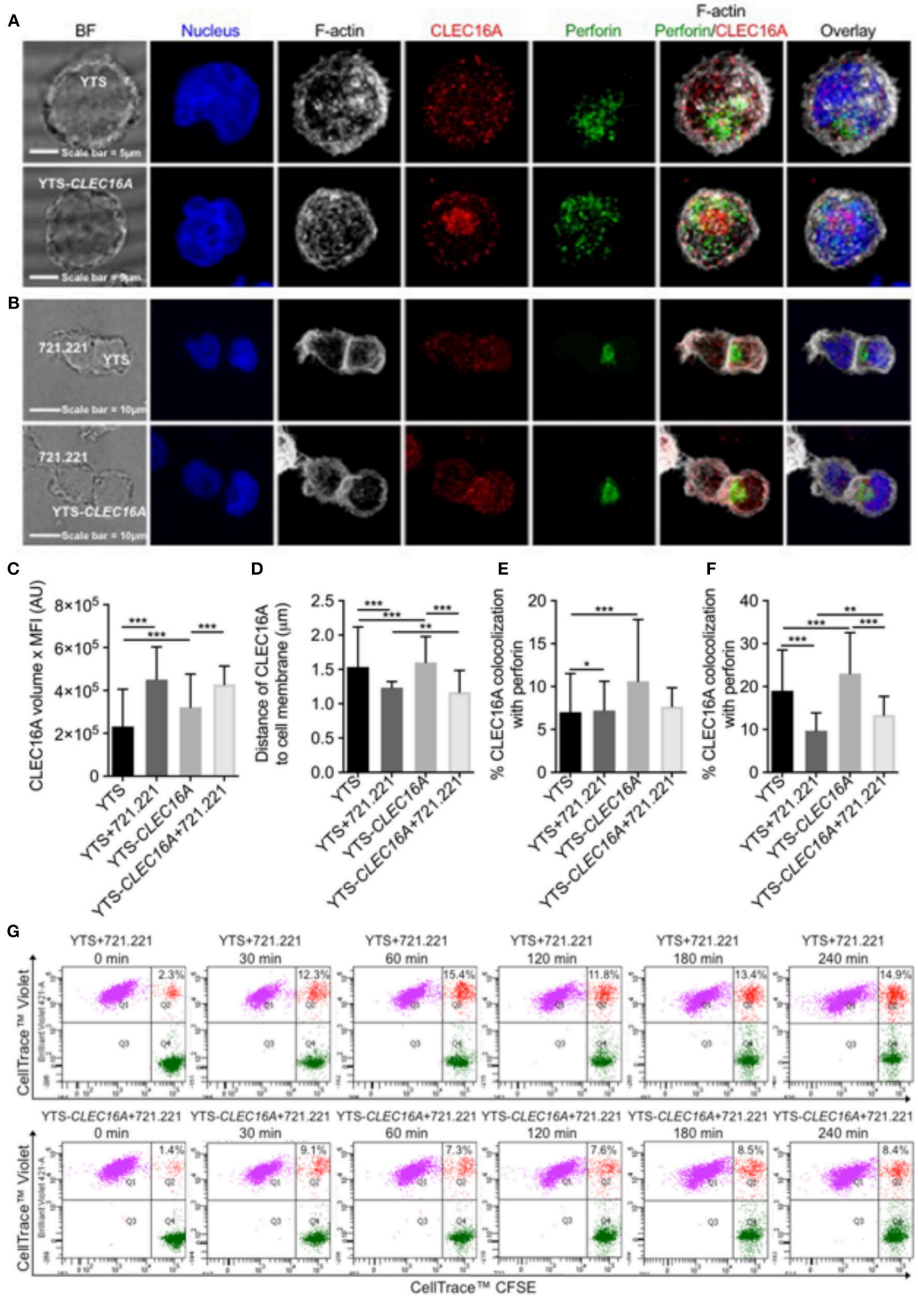
Overexpression restrains conjugate formation and migration in YTS-*CLEC16A*. **(A)** Representative confocal microscopy images of unconjugated YTS (top panel) and YTS-*CLEC16A* (bottom panel) cells demonstrating localization of CLEC16A. Images are z-projections, except the BF which is a single image taken at the plane of the glass; scale bar = 5 μm; BF = bright field. **(B)** Representative confocal microscopy images of conjugates formed between YTS and 721.221 (top panel) and YTS-*CLEC16A* and 721.221 (bottom panel). Effectors and targets were incubated at 37°C for a total of 30 min at an E:T ratio of 1:2. Scale bar = 10 μm. **(C)** Total amount of CLEC16A per-cell, **(D)** Mean distance of CLEC16A vesicles to cell membrane per cell, **(E)** Percent of CLEC16A vesicles colocalized with perforin vesicles, and **(F)** Percent of perforin vesicles colocalized with CLEC16A vesicles, for effectors alone or effectors in conjugate with 721.221 (after 30 min of conjugation). A total of 200–600 cells were analyzed for effectors alone and 15–25 cells for effectors in conjugate per condition in each experiment. Results show are mean ± SE of three independent experiments determined using an unpaired two-tailed Student's *t*-test; ^*^p < 0.05, ^**^p < 0.01, ^***^p < 0.001. **(G)** Effect of CLEC16A overexpression on conjugate formation. The percent of YTS and 721.221 conjugates formed in a time course experiment, shown in upper panel. The percent of YTS-*CLEC16A* NK cells bound to 721.221 targets in a time course experiment (bottom panel). The time series is representative of three independent repeats. Data represents means ± SE of three independent experiments. CLEC16A overexpression in YTS-*CLEC16A* results in decreased conjugate formation with 721.221 targets in comparison to parental YTS.

To address CLEC16A localization following NK/target cell interaction, effectors (YTS and YTS-*CLEC16A* cells) and target (721.221) cell conjugates were evaluated in confocal microscopy. Confocal images show that CLEC16A has cytoplasmic localization (Figures 3A,B). Quantitative analysis revealed that un-conjugated YTS-*CLEC16A* showed a significant increase in CELA16A volume in comparison to parental YTS. Upon conjugation for both cell types there was no significant difference. Both YTS and YTS-*CLEC16A* in conjugates exhibited a significant increase in CLEC16A in comparison to their respective unconjugated cells (Figure 3C).

We next analyzed the mean distance of CLEC16A vesicles to cell membrane per cell. The average distance between CLEC16A and cell membrane significantly decreased in YTS vs. YTS-*CLEC16A*, YTS-conjugate vs. YTS-*Clec16a* conjugate, YTS vs. YTS-conjugate and YTS-*CLEC16A* vs. YTS-*CLEC16A*-conjugate (Figure 3D). Upon conjugation, YTS-*CLEC16A* conjugates showed the highest decrease in the mean distance of CLEC16A vesicles to cell membrane (closer to synapse). Thus, CLEC16A vesicles migrate closer to the cell membrane upon conjugate formation (Figure 3D). We also calculated the % of CLEC16A vesicles co-localized with perforin vesicles and vice versa for the both effectors alone and the effectors in conjugate with 721.221 targets. Unconjugated YTS-*Clec16a* vs. YTS showed significant increases in % CLEC16A co-localized with perforin. However, only YTS in conjugates exhibited a significant decrease in % CLEC16A co-localized with perforin (Figure 3E). Similarly, % perforin co-localized with CLEC16A was significantly higher in YTS-CLEC16A in comparison to YTS. Upon conjugation % perforin co-localization significantly decreased for both cell types but was higher for YTS-*CLEC16A* cells (Figure 3F), suggesting that CLEC16A could play role in restraining granule release at the synapse.

Since CLEC16A expression affects NK cell cytotoxicity and CLEC16A migrates closer to the cell membrane upon formation of the immunological synapse, we examined whether decreased target killing results from decreased ability to form conjugates. We performed multi-color flow cytometry-based assay to study conjugate formation in time course experiment for both cell types (parental YTS and YTS-*CLEC16A*). Upon conjugation with 721.221 targets, both effectors rapidly formed conjugates. Overexpressing YTS-*CLEC16A* NK cells formed significantly fewer conjugates for all time points in comparison to YTS (Figure 3G; Supplementary Figure S1B). We also evaluated CD107a expression and we observed no significant differences between YTS and YTS-*CLEC16A* NK cell lines in terms of percent of conjugated NK cells expressing CD107a following stimulation with 721.221 targets (Supplementary Figure S1C). These results demonstrate that overexpression of *CLEC16A* restrains conjugate formation and may affect downstream NK cell function.

### CLEC16A Interacts With the Class C Vps-HOPS Complex and Modulates NK Cell Surface Receptors via Autophagy

**Figure 4:**
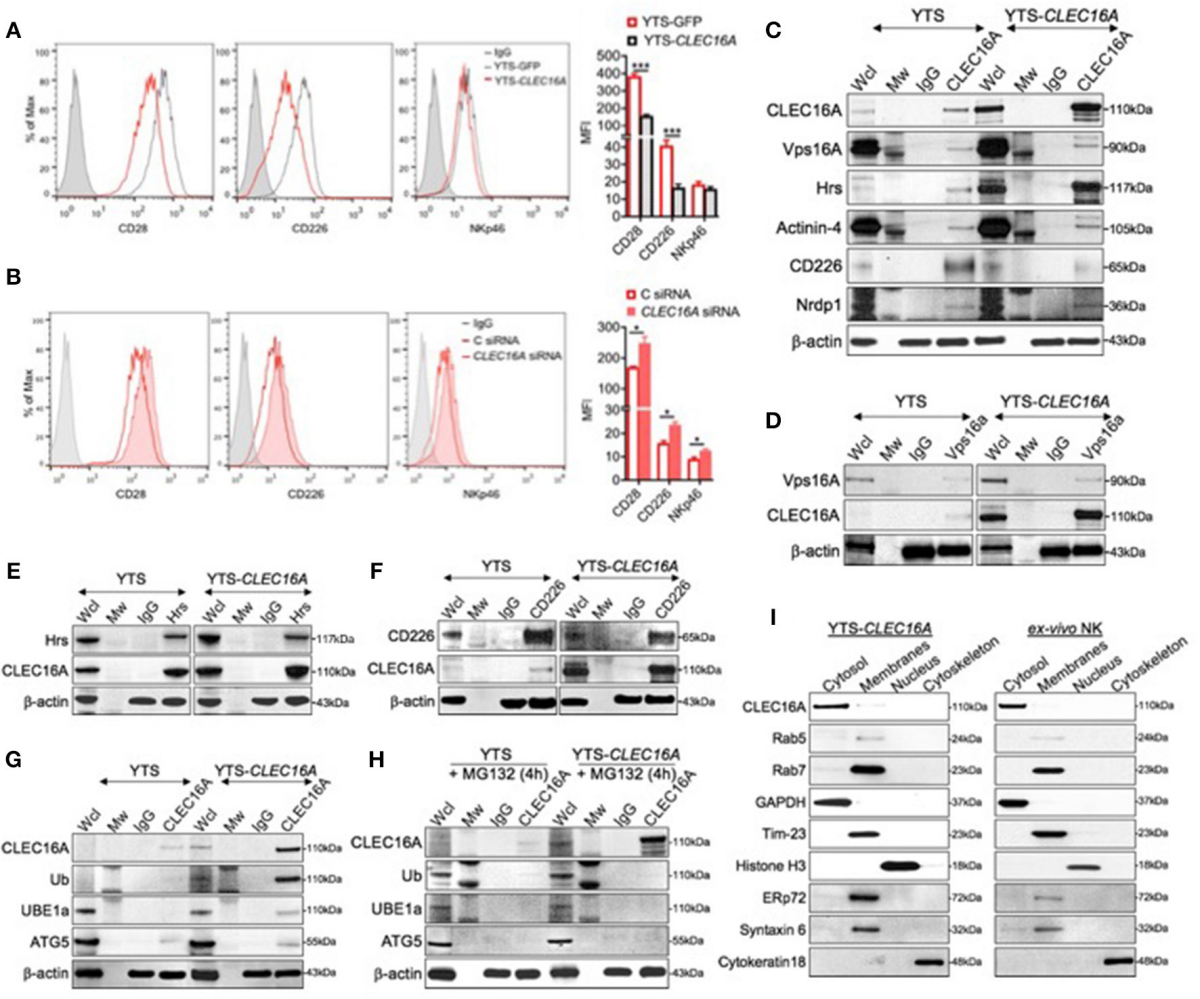
CLEC16A interacts with the class C Vps-HOPS complex, modulates NK cell surface markers expression and depicts cytosolic localization. **(A)** Representative histogram (left) and mean fluorescent intensities (MFI) of cell surface receptor CD28, CD226, and NKp46 in *Clec16a* overexpressing cells and YTS-GFP control (*n* = 3 repeats) by flow cytometry analysis. **(B)** Reversal of receptor expression in siRNA mediated CLEC16A knockdown. Representative histogram (left) depicts percentage decrease in expression of CD28, CD226, and NKp46 in control and *CLEC16A* siRNA nucleofected cells. MFI graph (right) depicts increase in expression of CD28, CD226, and NKp46 in YTS-*CLEC16A* overexpressing cells and YTS-GFP control (*n* = 3 repeats). ^*^P < 0.05, ^***^P < 0.001 (unpaired two-tailed Student's *t*-test). **(C)** CLEC16A IP-pull down and representative immunoblot analysis of CLEC16A, Vps16A, Hrs, Actinin-4, and CD226 and Nrdp1. Membranes were stripped and re-probed for β-actin as a loading control (bottom). **(D)** Representative reverse IP-pull down and immunoblot of VPS16A and CLEC16A in YTS and YTS-*CLEC16A*. **(E)** Representative reverse IP-pull down and immunoblot of Hrs and CLEC16A in YTS and YTS-*CLEC16A*. **(F)** Representative reverse IP-pull down and immunoblot of Hrs and CLEC16A in YTS and YTS-CLEC16A. **(G)** Representative CLEC16A IP-pull down and immunoblot of CLEC16A, Ub, UBE1a, and ATG5 in YTS and YTS-*CLEC16A*. **(H)** Representative CLEC16A IP-pull down and immunoblot of CLEC16A, Ub, UBE1a, and ATG5 in YTS and YTS-*CLEC16A* cells pretreated with proteasomal inhibitor MG132. Membranes were stripped and re-probed for β-actin as a loading control for all pull down experiments (bottom). **(I)** Representative immunoblot depicting cytosolic localization of CLEC16A in YTS-*CLEC16A* and *ex vivo* NK cell fractioned lysates. Membranes were stripped and probed for GAPDH as loading control for cytosolic fraction, Rab5 (early endosomes marker), Rab7 (late endosomes marker), Tim-23 (mitochondrial membrane fraction), Histone H3 (Nucleus), ERp72 (endoplasmic reticulum (ER), Syntaxin 6 (Golgi), and Cytokeratin 18 (cytoskeleton loading control).

In view of the above findings, we hypothesized that the attenuation of signaling in the context of *Clec16a* overexpression could be due to increased targeting of receptors for degradation via autophagy in lysosomes. Eight NK cell receptors that use a variety of proximal signaling molecules were studied in a flow-based assay: CD28, CD11a, CD226, NKp46 (CD335), NKp30 (CD337), CD16, CD18, and NKG2D (CD314). YTS-*CLEC16A* cells showed significant decreases in mean fluorescence intensity (MFI) of CD28 and CD226 activating receptors relative to YTS-GFP cells. No significant reduction in MFI for NKp46 was observed (Figure 4A). NKp30, CD16, NKG2D, and the adhesion markers CD11a and CD18 showed no significant differences in expression between the cell lines (data not shown). To further confirm the role of CLEC16A in the NK cell receptor modulation we used siRNA to mediate *CLEC16A* knockdown in YTS-*CLEC16A* cells. As expected, we observed a significant reversal in the expression of activating receptors with *CLEC16A* specific siRNA relative to cells treated with control siRNA: a significant increase in MFI for all three activating receptors was observed (Figure 4B). These results confirm the role of CLEC16A in NK receptor modulation.

In view of our *in vitro* NK cell receptor expression profile, and the previously established association of CLEC16A with the class C Vps-HOPS complex (13), we hypothesized that CLEC16A participates in the receptor modulation. To test this hypothesis, we performed a series of reciprocal co-immunoprecipitation (co-IP) experiments using YTS and YTS-*Clec16a* NK cells. CLEC16A immunoprecipitation experiments demonstrate that endogenous CLEC16A binds to Vps16a, ESCRT protein-Hrs, and Actinin-4 (Figure 4C). Reverse co-IP and immunoblot confirmed the association of Vps16A, a subunit of the class C Vps-HOPS complex (Figure 4D), and Hrs (Figure 4E) with CLEC16A. In contrast to co-IP in drosophila, where Hrs does not bind Ema (13), our co-IP experiments revealed an association between CLEC16A and Hrs (Figures 4C,E). It was reported earlier that Hrs/actinin-4/BERP/myosin V (CART [cytoskeleton-associated recycling or transport]) complex assembles in a linear manner and is required for efficient receptor recycling (21). To further strengthen the role of CLEC16A in regulation of receptor trafficking, we probed the CLEC16A pull down for CD226. We observed less CD226 from YTS-*Clec16a* pulldown in comparison to YTS (Figure 4C). IP performed with CD226; when probed for CLEC16A it showed more CLEC16A in overexpressing cells (Figure 4F). This may be because there is more CLEC16A in YTS-*CLEC16A* cells. The degree of association varies with the level of CLEC16A expression. YTS with low CLEC16A levels demonstrated a strong association with CD226; yet with high CLEC16A levels the association with CD226 decreases (Figure 4C). In YTS-*CLEC16A*, excess CLEC16A may be involved in degradation of CD226. Our findings demonstrate that CLEC16A regulates receptor trafficking via the CART complex in YTS and YTS-*CLEC16A* cells.

Hrs protein belongs to the endosomal-sorting complex required for transport (ESCRT)-0 (22). Many of ESCRT-dependent processes are tightly regulated by Ubiquitin (Ub), which serves as a lysosomal-sorting signal for membrane proteins targeted into multi-vesicular bodies (MVBs). Since we observed receptor downregulation in YTS-*CLEC16A* cells, we hypothesized that CLEC16A associates with the receptor cargo and targets them for degradation via autophagy, serving as a critical checkpoint, thus preventing hyperactivity of NK cells. To test this hypothesis, we performed CLEC16A IP in YTS and YTS-*CLEC16A* cells with IgG and CLEC16A antibody and probed for CLEC16A, ubiquitin (Ub), the ubiquitin-activating enzyme (UBE1a), and Atg5 (23). Parental YTS cells showed no CLEC16A ubiquitination or association with UBE1a and a low degree of association with Atg5. In contrast, in YTS-*CLEC16A* showed association with UBE1a and Atg5 (Figure 4G). This association was lost in YTS-*Clec16a* cells pretreated with a proteasomal inhibitor MG132 (Figure 4H).

In light of the above findings, we next addressed CLEC16A localization. We performed an immunoblot analysis on overexpressing YTS-*CLEC16A* NK cell line and *ex vivo* NK cells using Qproteome Cell Compartment Kit. In both cell types, CLEC16A was highly enriched in the cytosol fraction and was barely detectable in the membrane fraction, which included ER and Golgi. To verify CLEC16A localization to cytosol we specifically probed the membrane (using GAPDH as loading control) for proteins such as Rab5 (early endosomes marker), Rab7 (late endosomes marker), Tim-23 (mitochondrial membrane), Histone H3 (Nucleus), ERp72 (endoplasmic reticulum (ER), Syntaxin 6 (Golgi), and Cytokeratin 18 (cytoskeleton loading control) (Figure 4I). Our results demonstrate that CLEC16A is a cytosolic protein and modulates receptor trafficking via the CART complex in YTS and *ex vivo* NK cells. Taken together, these results suggest that CLEC16A modulates receptor turnover and expression via the C Vps-HOPS complex, CART and autophagy.

### CLEC16A Overexpression Promotes Autophagy and Knockdown Triggers Disrupted Mitophagy

**Figure 5:**
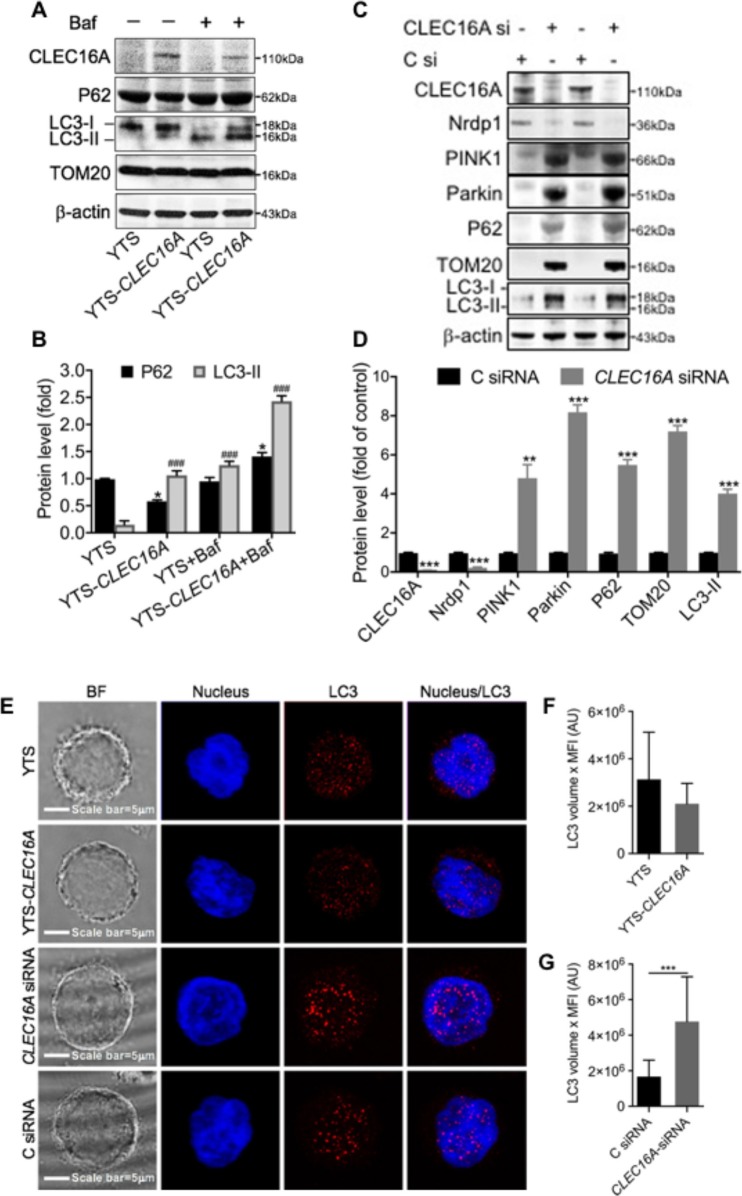
*CLEC16A* overexpression promotes autophagy and knockdown promotes disrupted mitophagy. **(A)** Representative Immunoblot depicting expression of ClEC16a, P62, LC3-I/II, and TOM20 from whole cell lysates of YTS, YTS-*CLEC16A* ± Bafilomycin. Membrane was stripped and reprobed for β-actin as loading control, shown in bottom panel. **(B)** Quantitation graph depicting P62 and LC3-II expression (*n* = 3 repeats). **(C)** siRNA mediated CLEC16A knockdown leads to disrupted mitophagy. Representative immunoblot blot for CLEC16A, Nrdp1, PINK1, Parkin, P62 TOM20, LC3-I/II expression from the whole cell lysates of YTS post 24 h of nucleofection with control and CLEC16A-siRNA. β-actin was probed as loading control. **(D)** Quantitation graph depicting CLEC16A, Nrdp1, PINK1, Parkin, P62 TOM20, LC3-I/II expression (*n* = 3 repeats). **(E)** CLEC16A knockdown leads to increased accumulation of autophagy marker LC3. Representative confocal microscopy images of YTS, YTS-*Clec16a*, Clec16a-siRNA and C siRNA cells depicting LC3 staining. Images are z-projections, except the BF which is a single image taken at the plane of the glass; scale bar = 5 μm; BF = bright field. **(F)** Total amount of LC3 per-cell in YTS and YTS-*Clec16a* and **(G)** Clec16a siRNA and C siRNA nucleofected YTS cells. A total of 20-33 cells were analyzed per condition in each experiment. Data represents means±SE of five independent experiments. ^*^P < 0.05, ^###^*p* < 0.001, ^**^P < 0.01, ^***^p < 0.001 (unpaired two-tailed Student's *t*-test).

We next investigated the effect of *CLEC16A* overexpression and knockdown on autophagy and mitophagy. We performed immunoblot analysis on YTS and YTS-*Clec16a* treated with and without bafilomycin and probed for two autophagy markers, P62/SQSTM and LC3-I/II. P62 is degraded by autophagy and accumulates upon inhibition of autophagy. LC3-II is a component of autophagosomes and is converted from LC3-I during autophagy. We found that at baseline, YTS-*CLEC16A* cells have a higher autophagic flux (lower p62) than YTS cells. Bafilomycin treatment inhibited basal autophagy. Immunoblot analysis showed a significant increase in p62 and LC3-II in YTS-*Clec16a* cells (Figures 5A,B). Outer mitochondrial membrane proteins such as TOM20 can be rapidly degraded by the proteasome under mitophagy inducing conditions (24). TOM20 showed no change, confirming absence of aberrant mitophagy in YTS, and CLEC16A overexpressing cells (Figure 5A).

We previously published that pancreatic CLEC16A interacts with E3 ubiquitin ligase Nrdp1 and plays regulates mitophagy via the Nrdp1/Pink1/Parkin pathway. We hypothesized that reduced expression of CLEC16A in NK cells would lead to disrupted mitophagy via Nrdp1/PINK/Parkin pathway. To test this hypothesis, we evaluated the effect of CLEC16A knockdown in YTS cells on mitophagy. siRNA mediated CLEC16A knockdown in YTS cells results in a significant decrease in Nrdp1 expression with CLEC16A knockdown. Immunoblot analysis showed a significant up-regulation of mitophagy proteins PINK1, Parkin, and TOM20 (Figures 5C,D). P62 and LC3-II significantly increased in knockdown compared to control. P62 is degraded by autophagy and accumulation of P62 suggests disrupted autophagy/mitophagy. We also performed confocal microscopy in YTS, YTS*-Clec16a*, and YTS cells nucleofected with *CLEC16A* siRNA and Control siRNA to study LC3 and obtain additional support for a role for CLEC16A in autophagy (Figure 5E). Total LC3 showed no difference between YTS and YTS-*Clec16a* cells (Figure 5F). CLEC16A KD in YTS cells resulted in a significant upregulation of LC3 (Figure 5G). Thus, we conclude that CLEC16A overexpression promotes autophagy whereas knockdown of CLEC16A induces disrupted mitophagy.

### *Clec16a Knockout* Leads to Increased Cytotoxicity in UBC-Cre-*Clec16a*^loxP^ Mice via Upregulation of NK Activating Receptors

To investigate the role of CLEC16A in NK cells we used our UBC- Cre-*Clec16a*^loxP/loxp^ mice, a whole-body inducible *Clec16a knockout* (KO) (19). Adult mice (10 weeks) were treated with tamoxifen (TAM) to induce the *Clec16a KO*. Control mice received an equal volume of vehicle (corn oil). We activated Cre recombinase in a wide range of tissues and blood upon TAM treatment. Cre recombination was confirmed by PCR using DNA isolated from whole blood. After removal of exon 3 in TAM treated mice we observed an 146 bp band in comparison to a 618 bp in control DNA. We confirmed the KO at the protein level in splenocytes from TAM treated (KO) mice by Western blot (Supplementary Figures S2A–C).

**Figure 6:**
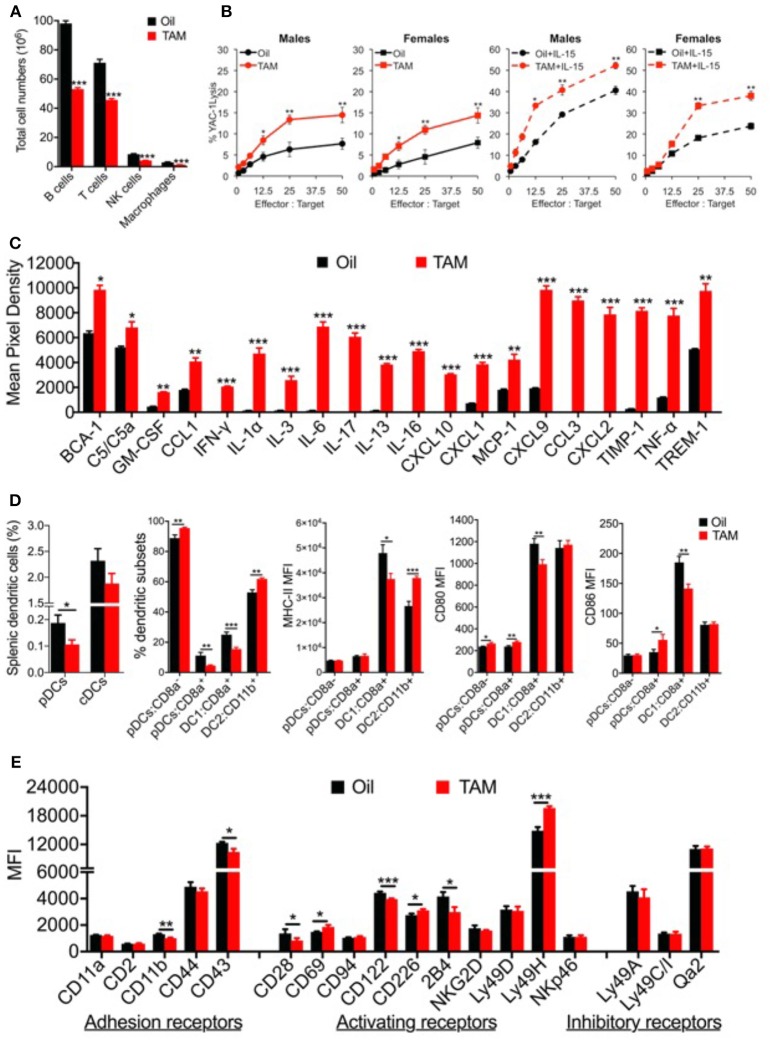
*Clec16a* knockout mice depicts altered immune cell populations, increased splenic NK cell cytotoxicity, upregulated cytokine and chemokine secretion and an imbalance in dendritic cell subsets with altered receptor expression. **(A)** Reduced spleen cell numbers and altered splenic Immune cell population in *Clec16a* KO (TAM) mice (*n* = 12 mice per group). ^*^P < 0.05, ^**^P < 0.01, ^***^P < 0.001 (unpaired two-tailed Student's *t*-test). **(B)** Cytotoxicity of resting and mrIL-15 (100 ng/ml) activated male and female murine splenocytes against ^51^Cr-labeled YAC-1 targets. Results show mean ± SE of three independent repeats. ^*^P < 0.05, ^**^P < 0.01, ^***^P < 0.001 (unpaired two-tailed Student's *t*-test). **(C)** The representative graph is quantification of cytokines and chemokine from plasma of Control (Oil) and *Clec16a* KO (TAM) mice using the Mouse Cytokine Array panel. Data represents means ± SE of three independent experiments. ^*^P < 0.05, ^**^P < 0.01, ^***^P < 0.001 (unpaired two-tailed Student's *t*-test). **(D)** Percent splenic dendritic cells (pDCs and cDCs), percent DC subsets, MHC-II, CD80, and CD86 MFI on pDCs, cDCs, and subsets from *Clec16a* KO and control mice analyzed by flow cytometry (*n* = 10 mice per group). Data represents means ± SE of three independent experiments. ^*^P < 0.05, ^**^P < 0.01, ^***^P < 0.001 (unpaired two-tailed Student's *t*-test). **(E)** MFI quantification graph depicting adhesion, activating and inhibitory receptors expressed on NK cells of control and *Clec16a* KO mice. Data represents means ± SE of three independent experiments (*n* = 12 mice per group). ^*^P < 0.05, ^**^P < 0.01, ^***^P < 0.001 (unpaired two-tailed Student's *t*-test).

We assessed the splenic immune cell populations by flow cytometry using a gating strategy as shown in Supplementary Figure S3. *Clec16a* KO mice show significant reduction in total numbers of B, T, NK cells, and macrophages (Figure 6A). Total splenocyte numbers and % immune cell types redistribution is shown in Supplementary Figure S3C. We recently demonstrated that TAM treated (*Clec16a* KO) mice show atrophy of thymus, spleen and significant reduction in total splenocytes numbers (19). We next evaluated NK cytotoxicity in the splenocytes of TAM treated and control mice, with and without rmIL-15 activation post 48 h in a standard 4-h ^51^Cr release assay. Resting murine NK cells are minimally cytotoxic against their targets and activation is required to induce cytotoxicity with IL-15. At an effector: target ratio of 50:1, resting control splenocytes exhibited only 7.6 ± 1.8% (males) and 7.9 ± 1.3% (females) of YAC-1 killing. In contrast, resting murine NK cells from TAM (*Clec16a* KO) male (14.5 ± 1.8%) and female (14.3±1.8%) mice demonstrated a significant increase in cytotoxicity. Cytotoxicity dramatically increased after splenocytes were activated with IL-15. At an identical effector: target ratio of 50:1, control splenocytes demonstrated 40.6 ± 1.0% (males) and 23.7 ± 1.4% (females) cytotoxicity, respectively. TAM treated (*Clec16a* KO) mice exhibited even greater enhancement in cytotoxicity, 52.2 ± 2.5% (males) and 39 ± 2.1% (females). Loss of CLEC16A resulted in a significant increase in killing of YAC-1 targets by murine NK cells despite a decrease in numbers (Figures 6A,B).

We next examined the inflammation profile in plasma from control and *Clec16a* KO mice using Mouse Cytokine Array panel (Supplementary Figure S4). *Clec16a* KO mice showed a significant upregulation of BCA-1, complement C5/C5a, cytokines (IFN-γ, IL-1α, IL-3, IL-6, IL-17, IL-13, IL-16, TNF-α), chemokines and chemokine receptors (GM-CSF, CCL1, CXCL10, CXCL1, MCP-1, CXCL9, CCL3, CXCL2) and TIMP-1 and TREM-1 proteins in comparison with control mice (Figure 6C). Collectively these results demonstrate that CLEC16A plays a critical role in constraining major functions of NK cells and production of inflammatory regulators.

The bi-directional cross-talk between NK and DCs during innate and adaptive immune responses has been well-elucidated. In light of the observed impaired NK cell functions shown above, we decided to evaluate the role of *Clec16a* KO on DCs. Splenic dendritic cells from *Clec16a* KO and control mice were analyzed by flow cytometry. DCs were identified as described in methods using the gating strategy shown in Supplementary Figure S5. We observed a significant decrease in the % of plasmacytoid dendritic cells (pDCs), but no change was detected in the classical dendritic cell (cDCs) population. Further evaluation of DC subsets revealed a significant increase for pDC-CD8a^−^ and decrease for pDC-CD8a^+^. For cDCs subsets we observed a significant decrease in DC1:CD8a^+^ and a significant increase for DC1:CD11b^+^. We also evaluated MHC-II, CD80, and CD86 expression in dendritic cell subsets. The pDC-CD8a^−^ subset showed a significant increase in CD80. The pDC-CD8a^+^ set showed a significant increase in both CD80 and CD86. The DC1 subset showed a significant decrease in expression for MHC-II, CD80, and CD86. In contrast, the DC2 subset showed a significant increase for MHC-II only (Figure 6D). Overall, these results demonstrate a unique imbalance of dendritic cell subsets in *Clec16a* KO mice.

As CLEC16A contains a c-type lectin domain, with no transmembrane region, we propose that CLEC16A functions intracellularly to retain other c-type lectin-containing receptors that would normally enable NK cell activation. To investigate if reduction of CLEC16A changes the cell surface expression of NK receptors and if this is the valid mechanism, we evaluated 18 NK cell receptors expressed in splenic NK cells of control and *Clec16a* KO mice in a flow-based assay. The gating strategy is shown in Supplementary Figure S6. We observed a significant decrease in MFI in two adhesion receptors (CD11b and CD43). Among activating receptors investigated, CD28 and CD122 showed a significant decrease, and CD69, CD226, and Ly49H showed a significant increase in MFI. The KIR family do not contain c-type lectin domains; therefore, we anticipate that this mechanism would not affect the expression and function of the major NK cell inhibitory system. We saw no change for inhibitory receptors ly49A, Ly49C/I, and Qa2 (Figure 6E).

**Figure 7:**
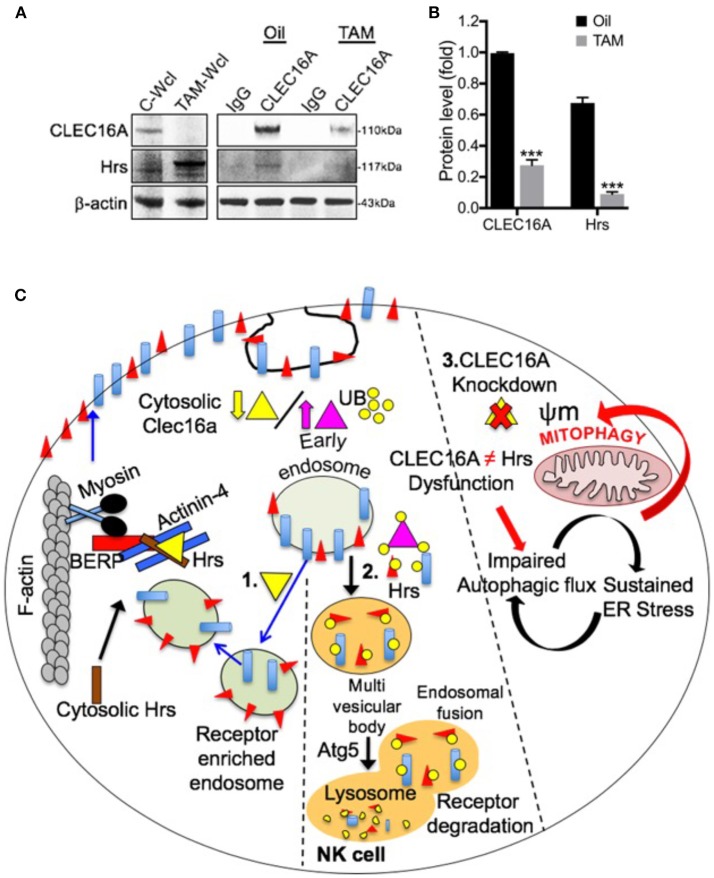
Loss of CLEC16A-Hrs interaction in *Clec16a* KO mice splenocytes and schematic diagram depicting multifaceted CLEC16A: receptor recycling and critical regulator of autophagy/mitophagy. **(A)** CLEC16A IP-pull down and representative immunoblot depicting expression of CLEC16A and Hrs from control (Oil) and *Clec16a* KO (TAM) mice. Membranes were stripped and re-probed for β-actin as a loading control. **(B)** Quantitation graph depicting CLEC16A and Hrs expression (*n* = 3 repeats). ^***^P < 0.001 (unpaired two-tailed Student's *t*-test). **(C)** NK cell requires a fine balance in the amount of CLEC16A protein. On the left side of figure (1), there is a normal level of endogenous CLEC16A that controls receptor trafficking by participating in endosome recycling. Binding of CLEC16A to the Hrs/Actinin-4/BERP/Myosin V [CART(cytoskeleton associated recycling or transport)] complex supports transport of receptor enriched endosomes to the cell surface membrane. The amount of this protein could be a checkpoint for the NK cell. When overexpressed [middle panel (2)], CLEC16A itself is getting ubiquitinated. There is an increased association of the ubiquitinated CLEC16A with the CART complex and the efficient receptor recycling is interrupted. In this situation the receptors will be targeted for degradation via autophagy. Thus, receptor signaling will be attenuated when CLEC16A is overexpressed. On other hand, CLEC16A is known to stabilize and prevents the proteosomal degradation of Nrdp1 limiting recruitment of Parkin to the mitochondrial surface and also promotes autophagosome-lysosome fusion during the terminal steps of mitophagy. Loss of CLEC16A will lead to an accumulation of unhealthy mitochondria due to disrupted mitophagy [right (3)].

We further investigated the CLEC16A-Hrs interaction in *Clec16a* KO mice to elucidate its role in receptor recycling and turnover. In contrast to results from over-expression, CLEC16A-Hrs interaction was decreased/lost in *Clec16a* KO mice creating an imbalance in receptor turnover (Figures 7A,B). A schematic diagram for multifaceted CLEC16A: receptor recycling and critical regulator of autophagy/mitophagy is depicted in Figure 7C. Thus, in mice, CLEC16A knockout modulates expression of NK cell surface receptors and functions, which is similar to what we observed in siRNA mediated CLEC16A knockdown in YTS NK cells and in opposite direction from the overexpression results, supporting the overall role of CLEC16A in restraining proinflammatory functions and cytolytic activity. Our study underscores a critical role of CLEC16A in YTS and *Clec16a* KO mice. We conclude that a delicate balance of CLEC16A activity appears to be necessary for cellular homeostasis.

## Discussion

CLEC16A is a key player in the pathogenesis of multiple autoimmune diseases (1–5, 7, 8, 10, 11, 25–28), suggesting the contributions of CLEC16A may be attributed to modulation of shared immune pathways. As a consequence, CLEC16A has become an attractive candidate for functional studies to explore the pathogenic mechanisms involved and potential therapeutic focus for autoimmune disorders. While, CLEC16A is clearly associated with multiple autoimmune diseases, little is known about its function. The Drosophila ortholog of *CLEC16A, ema*, was first characterized as an endosomal membrane protein required for endosomal trafficking and maturation. Expression of the human ortholog of *ema*, CLEC16A, rescued the drosophila null mutant, demonstrated conserved function of the protein (13). Later, the same group reported that ema facilitates the growth of autophagosomes (14). Another study elucidated the dynamic expression changes and localization of CLEC16A in lipopolysaccharide (LPS)-induced neuroinflammatory processes in adult rats (29). Investigation of CLEC16A involvement in T-cell co-stimulation and activation in human lymphoblastoid cell lines (LCLs) showed that *CLEC16A* knockdown does not affect T cell activation or proliferation following co-culture with KD or control LCLs (30).

Data in mice with a pancreas-specific deletion of *Clec16a* show that CLEC16A is required for normal glucose-stimulated insulin release through its effect on mitophagy (15). The clinical significance of this work is supported by the findings in patients with the *CLEC16A* T1D risk variant, rs12708716-G, who have reduced expression of CLEC16A in islets and attenuated insulin secretion. A recent study reported Clec16a, Nrdp1, and USP8 function in a ubiquitin-dependent protein complex to regulate β-cell mitophagy (31). These findings however, do not address the role of CLEC16A in immune or immune cell subsets, a critical component of autoimmunity.

We recently demonstrated a functional link between CLEC16A and disrupted mitophagy in immune cells and have now shown that incomplete mitophagy predisposes the *Clec16a* KO mice to a cascade of inflammatory reactions resulting in inflammation (19). This work was designed to better define the role of CLEC16A in NK cells, inflammation, and autoimmune disorders. In our functional studies of CLEC16A we initially utilized the NK cell lines NKL and YTS as they are homozygous for the protective (A/A) and non-protective (G/G) alleles (rs2903692-A), respectively. NKL showed high CLEC16A expression and low cytotoxicity in comparison to YTS as anticipated (32). Since *CLEC16A* SNPs were associated with protection from type-1 diabetes, some of the highest CLEC16A expression was identified in NK cells, and finally, given previously reported associations of NK cells with multiple autoimmune diseases, we can assume that defining the role of CLEC16A in NK cells may uncover novel insight into disease pathogenesis. As NK cells are both cytotoxic and producers of cytokines, they have the potential to contribute both directly and indirectly to tissue and organ damage at pathological sites in autoimmune disorders and may reveal an important insight into the pathogenesis of autoimmunity.

Our expression analysis of CLEC16A at the protein level in human immune cell types revealed highest expression in B cells followed by NK and T cells. These findings confirmed the expected pattern of CLEC16A expression based on the available microarray data (33, 34). It also suggests that protein expression and action may be relevant in these cell types. Recently, we reported a functional link between CLEC16A and disrupted mitophagy in immune cells, which showed that incomplete mitophagy predisposes the *Clec16a* KO mice to inflammatory reactions and inflammation (19). Our findings in the current study unveil the role of CLEC16A in restraining major NK cell functions: cytotoxicity, cytokine release and DC maturation. The overexpression approach resulted in a significant reduction of 721.221 targets killing and IFN-γ production. To further assure that CLEC16A acts to restrain NK cell cytolytic activity we assessed the effects of siRNA mediated knockdown (KD). As anticipated, reducing the CLEC16A expression at the protein level resulted in increased NK cell killing. Since the level of CLEC16A expression affected NK cell cytolytic activity, we asked whether decreased target cell killing is attributed to decreased ability to form conjugates. NK cells, overexpressing CLEC16A, showed a significant decrease in conjugate formation in comparison to endogenous control, which was evident as early as 30 min and remained constant over the time course. Further, we evaluated CD107a expression, an established functional marker for NK cell activity (35), and found no difference in the CD107a expression on conjugates formed over 0 to 240 min with 721.221 targets between parental YTS and Clec16a NK cells.

Cytolysis mediated by the YTS NK cell line depends on signals from two surface receptors: CD28 and CD11a (36). The most abundantly expressed adhesion marker CD11a (36) showed no difference in YTS and YTS-*Clec16a* cells. We found a significant decrease in MFI of CD28 and CD226 in YTS-*Clec16a* cells in comparison to parental YTS. Since we did not see any defect in adhesion markers, we propose that CLEC16A could have a role in activation-induced-signaling in NK cells, after seeing decreased expression of activating receptors. Substantial evidence indicates that NK cell function relies on receptor-ligand interaction (17).

Previous studies suggest that CLEC16A is a membrane-associated protein (13, 15, 30). Our results show that CLEC16A is a cytosolic protein in NK cells and this finding is in concordance with its function to restrain the cytolytic activity and cytokine release. In search of potential candidates interacting with CLEC16A we performed STRING analysis. STRING predicted CD226 and Vps16A as first-degree potential partners of CLEC16A. in the presence of CD226 crosslinking in NK cells overexpressing CLEC16A, the NK cells showed a significant decrease in IFN-γ production. CD226 is an activating receptor that is expressed on the majority of immune cells including NK cells, T-cells, monocytes and platelets and is involved in intercellular adhesion. It can also stimulate NK cell mediated cytotoxicity and the secretion of proinflammatory cytokine IFN-γ (37). The CD226 gene is a confirmed susceptibility gene for T1D (38, 39) and other autoimmune disorders (40, 41). To investigate the expression and degree of association between CD226 and CLEC16A, we performed reciprocal co-IP experiments, confirming an association between CLEC16A and CD226. We found that cells with low CLEC16A levels show a strong association with CD226, however, with an increase in CLEC16A expression the association with CD226 is lost.

Association between CLEC16A ortholog ‘ema’ and C Vps-HOPS complex has been shown previously in drosophila (14). The class C Vps-HOPS complex participates in many aspects of receptor mediated endocytosis and subsequent trafficking through the endosomal pathway. Signaling can be attenuated by targeting active receptors for degradation in lysosomes or potentiating by trafficking receptors to signaling endosomes (42). Vps16A is a subunit of the class C Vps-HOPS complex, which plays a role in vesicle mediated protein trafficking to lysosomal compartments and in membrane docking/fusion reactions of late endosomes or lysosomes (43, 44). Our reciprocal co-immunoprecipitation experiments in NK cell lines confirmed the association between CLEC16A and Vps16A and revealed that the degree of association depends on the level of CLEC16A expression. NK cells with low level of CLEC16A showed more association with Vps16A; with overexpression of CLEC16A the association with Vps16A decreases. A recent study reported that CLEC16A has intrinsic E3 ligase activity, thereby possesses the ability to self-ubiquitinate and functions as a ubiquitin-dependent tripartite complex (Clec16a-Nrdp1-USP8) to regulate β-cell mitophagy (31). We show that CLEC16A over-expression promotes autophagy and knockdown triggers disrupted mitophagy. Although different models for CLEC16A function are possible, our data from the overexpression system and knockout mouse model suggest that CLEC16A has intrinsic E3 ligase activity and possesses the ability to self-ubiquitinate, and modulate receptor turnover and expression via the C Vps-HOPS complex, CART and autophagy.

Importantly, our whole-body inducible *Clec16a* KO murine model demonstrates that post-developmental *Clec16a* knockout results in altered splenic immune cell population. Although a significant decrease in the percentage of NK cells is observed in *Clec16a* KO mice, loss of CLEC16A specifically attributes to increased killing of targets for all genders with and without NK cell activation. Even the resting murine NK cells from *Clec16a* KO mice demonstrated a two-fold increase in cytotoxicity. After activation with mrIL-15, the cytotoxicity dramatically increased: *Clec16a* KO mice exhibited significantly higher cytotoxicity in comparison to control mice.

Altered immune cell populations, increased killing, upregulated cytokines/chemokine secretion, or an imbalance in DC subsets observed under previously established disrupted mitophagy settings, may lead to excessive cell death and tissue destruction and may reflect the inflammatory mechanism utilized during the development, progression, and pathogenesis of various autoimmune and inflammatory diseases. Dead cells constitute a source of novel antigens that can further provoke the autoimmune response. The abundance of proinflammatory cytokines including IL-6, TNF-α, GM-CSF, and chemokines is crucial for pDC and cDC activation and function (45). In recent years, the essential role of bi-directional cross-talk between NK and DCs during immune responses has been more clearly elucidated (45), however, further investigation surrounding NK/DC crosstalk is warranted. Cross-talk results in the development of an efficient innate immune response through DC-mediated NK cell activation, and a potent adaptive immune response through NK-mediated DC editing and maturation. The derailed functional link between CLEC16A and NK cells, that we describe here, may further explain the immune dysregulation under previously established disrupted mitophagy conditions in immune cells (19) and the risk of autoimmunity associated with specific CLEC16A variants.

In summary, the autoimmune disorder susceptibility gene *CLEC16A* restrains NK cell function in the YTS NK cell line and *Clec16a* knockout mice. Thus, our study underscores a critical role of CLEC16A in NK cells under previously established disrupted mitophagy conditions in immune cells.

## Materials and Methods

### Cell Culture

The YTS, 721.221, K562, and YAC-1 cell lines were grown at 37°C in 5% CO_2_ in complete RPMI medium (Gibco), where “complete” indicates supplementation with 10% FBS (Thermo Scientific), L-glutamine, non-essential amino acids, sodium pyruvate, HEPES, and penicillin-streptomycin (all from Gibco). The NKL cell line was grown in Myelocult-5100 culture medium (StemCell Technologies) containing 100 U/ml human recombinant IL-2. The YTS-*CLEC16A* cell line was grown at 37°C in 5% CO_2_ in complete RPMI medium with G418 (1.6 g/L, Mediatech). Where specified, cells were treated *in vitro* with 100 ng/ml PMA plus 1 μg/ml ionomycin (PMA+I) or with plate-bound anti-CD28 or anti-NKp30 (5 μg/ml for each antibody). Human *ex vivo* NK cells purchased from the University of Pennsylvania Human Immunology Core Facility were immediately used in knock down and cytotoxicity experiments. Murine NK cells from splenic cell suspensions were negatively selected using EasySep Mouse NK cell Enrichment Kit, following the manufacturer's instructions (StemCell).

### Molecular Cloning and Generation of YTS-CLEC16A Cell Line

A full-length Human cDNA clone of *CLEC16A* (encoding amino acids 1–1,053) was purchased from OriGene Technologies. EcoR I and Bgl II restriction sites were added via overlapping PCR amplification to allow cloning into pCR-Blunt II-TOPO (Invitrogen) and, subsequently, formation of the bicistronic retroviral expression vector pMx-IRES-GFP (46). Vectors were packaged into ecotopic retroviruses and used to infect YTSeco cells, as previously described (47). Transduced YTSeco cells were identified through GFP expression and sorted by FACS to create cell culture stably overexpressing *CLEC16A* (YTS-*CLEC16A*).

### Flow Cytometry

Transduced YTSeco cells were identified through GFP expression and sorted by FACS to create cell culture stably overexpressing *CLEC16A*. To evaluate the number of conjugates formed in 0, 30, 120, 180, and 240 min, effector cells (YTS and YTS-Clec16a) labeled with CellTrace^TM^ CFSE Cell proliferation Kit and target cells (721.221) labeled with CellTrace^TM^ Violet Cell proliferation Kit (Invitrogen) were combined in a 1:2 ratio and incubated in the presence of anti-human Alexa Fluor 647 conjugated CD107a antibody (BioLegend). Cells were fixed by adding 300 μl ice cold 2% paraformaldehyde solution to prevent further conjugate formation. Cell-associated fluorescence was assessed with a LSRFortessa flow cytometer (BD Pharmingen) and analyzed with FlowJo software (Tree Star).

To analyze the surface receptor phenotype in YTS-GFP and YTS-*CLEC16A* NK cells, a total of 1x10^6^ cells were labeled with anti-human PE-conjugated CD28, CD11a (LFA-1), CD226 (DNAM-1), NKp46 (CD335), NKp30 (CD337), CD16, CD18, NKG2D (CD314), NKp44 (CD336) and IgG isotype antibodies (BioLegend). After incubation, cells were fixed and analyzed. To assess the mitochondrial membrane potential, YTS-GFP and YTS-*CLEC16A* NK cells were stained with MitoTraker® Deep Red FM (31.3 nM) and analyzed by FACS. Cell-associated fluorescence was assessed with a FACSCalibur flow cytometer (BD Pharmingen) and analyzed with FlowJo software.

For flow cytometric analysis of murine splenic cells, spleens were harvested, and cell suspensions were prepared by dicing spleens with a razor blade and gently pressing through the 70 μm nylon cell strainer (Corning) with the plunger end of a 1 ml syringe in RPMI-40 (Invitrogen). Cells were washed in staining buffer (Ca^+2^ and Mg^+2^ free PBS containing 2% heat-inactivated fetal bovine serum and 5 mM EDTA), resuspended and counted using a Cellometer Auto X4 cell counter (Nexcelom Bioscience). Briefly, 2 × 10^6^ cells per tube were stained with different sets of antibodies as described below. Cell viability was assessed by incubation in the Live/Dead Blue dye (Invitrogen) (1:500) and dilution in Ca^+2^ and Mg^+2^ free PBS for 10 min in the dark at room temperature (RT). For all experiments, cells were incubated in 0.5 μg Fc Block (BD Biosciences) for 10 min at RT. Surface staining was performed in the dark for 30 min at 4°C in staining buffer. Cells were then washed twice with staining buffer followed by fixation in 2% paraformaldehyde. A comprehensive list of surface markers for all flow cytometry experiments is shown in Supplementary Table S1.

For identification of immune cell subsets in mouse spleen, the following anti-mouse antibodies were used: BV421™ CD45R/B220, PE/Cy7 CD3ε, PE CD49b, APC F4/80, BV711™ CD11b/Mac-1, FITC Ly-6G/Ly-6C (Gr-1) and FITC TER-119. For a phenotypic analysis of NK cells in splenic single-cell suspensions the following anti-mouse antibodies were used: PE CD49b, PE/Cy7 CD3ε, BV 421™ CD19, FITC CD94, FITC Ly-49A, AF®647 Qa-2, AFr®647 CD244.2 (2B4 B6 Alloantigen), AF®647 CD226 (DNAM-1), BV711™ CD335 (NKp46), FITC Ly49D Antibody, AF®647 Ly49H, AF® 700 CD69, FITC Ly-49C, and Ly-49I, BV421 CD122, BV711 CD314 (NKG2D), BV421™ CD44, FITC CD2, APC CD43, BV421™ CD11b, FITC CD11a, and APC CD28. For immunophenotyping of dendritic cell subsets in mouse spleens, the following anti-mouse antibodies were used: BV421™ CD45R/B220, BV510™ CD8a, BV605™ CD11c, BV711™ CD11b, AF®488 I-A/I-E, PE CD80, PerCP/Cyanine5.5 CD317 (BST2, PDCA-1) and APC/Cy7 CD86. For flow cytometry immunophenotyping experiments, cells were acquired on an LSR Fortessa or LSR II cytometer (both from BD Immunocytometry Systems). Compensation was performed at the beginning of each experiment using single color controls prepared from a splenocyte mixture for all mice used in the experiment. Compensation matrices were calculated and applied using BD FACS Diva software (BD Biosciences). Data were analyzed using FlowJo (Tree Star). Fluorescence minus one (FMO) controls were used for gating analyses to distinguish positively from negatively staining cell populations. Biexponential transformation was adjusted manually where necessary.

### Quantitative Real-Time PCR (qPCR)

Total RNA was isolated with Trizol reagent (Invitrogen) following RNA purification using the RNeasy Mini Kit (Qiagen) and converted to cDNA by High Capacity RNA-to-cDNA Kit (Applied Biosystems), according to the manufacturer's protocols. An assay comprising known human and murine CLEC16A RNA transcripts as well as control genes (β-Actin and HRPT1) were measured by real time PCR on a ViiA™ 7 Real Time PCR System, using predesigned 20X FAM-MGB TaqMan gene expression assays available from Applied Biosystems. All assays had primers covering exon-exon boarders to avoid DNA contamination. Triplicates were used for all samples included in the experiment. All PCR runs were performed on ViiA™ 7 Real Time PCR System using ViiA7 RUO software v1.2.2 (Life Technologies).

### Western Blot

For Western blot analysis, cell lysis was performed with NP40 lysis buffer (Invitrogen). The lysates were electrophoresed on 4–12% NuPAGE Bis-Tris gels in MOPS SDS running buffer and transferred onto nitrocellulose membranes (Invitrogen). The membranes were blocked in 3% BSA and incubated with indicated primary antibodies where specified: CLEC16A, PINK1 (Abgent), GFP, Nrdp1 (Novus Biologicals), TOM20 (ProteinTech), Parkin, p62/SQSTM1, LC3 I/II, (Santa Cruz). For analysis of autophagy progression, YTS-*CLEC16A* cells were cultured overnight in regular medium (RPMI with G418) or in RPMI without amino acids and serum containing 100 nM Bafilomycin A. Cell lysates were subjected to immunoblot analysis using anti-LC3, P62, TOM20, and β-actin antibodies were specified. The membranes were washed and incubated with a respective secondary antibody. Bound antibody was detected with WesternBright ECL chemiluminescence detection system (Advansta). Membranes were stripped and re-probed with mouse anti-β-Actin mAb (Abcam) as a loading control. Band intensities were measured using Image J software (NIH Shareware). Image J (National Institutes of Health) was used for all quantitative analysis of western blot. Briefly, films were scanned in gray scale mode at 300 DPI and saved in TIFF format. Using the Image J software, we obtained a histogram of each band and got an analysis of the area under each peak for both; proteins of interest and loading control.

### Subcellular Fractionation Experiments

5 × 10^6^ YTS-*CLEC16A* NK cells and *ex vivo* NK cells were used in Qproteome Cell Compartment Kit according to the manufacturer's instructions (Qiagen). Ten micrograms of protein per fraction was used for western blot. The membranes were probed for CLEC16A (Abgent), Rab5, Rab7, GAPDH, Tim-23, Histone H3, ERp72, Syntaxin 6 (Cell Signaling), and Cytokeratin 18 (Abcam).

### Co-immunoprecipitation

5 × 10^6^ YTS-*CLEC16A* and YTS cells were lysed in ice cold IP-lysis buffer. After centrifugation at 13,000 rpm for 10 min at 4°C, supernatants were pre-cleared with 50 μl of agarose G beads (Invitrogen) and IgG for 45 min at 4°C. The pre-cleared lysates were incubated with CLEC16A, Vps-16A, CD226, Hrs, Actinin-4, Nrdp1, IgG (Millipore) antibodies for 1 h at 4°C and then with 50 μl of agarose G beads for an additional 1 h at 4°C. Immune complexes were washed three times, dissolved in SDS sample buffer, electrophoresed, and transferred onto nitrocellulose membranes (Invitrogen). Western Blot analysis was performed as described above. Membranes were probed for CLEC16A (Abgent), Vps-16A (Santa Cruz), CD226, Hrs, and Nrdp1 (Novus BioLogicals). For CLEC16A in murine splenic cells, spleens were harvested and cell suspensions were prepared by dicing spleens. 250 × 10^6^ splenocytes from control and knockout mice were lysed in ice cold IP-lysis buffer and IP pull down was performed as described above. Membranes were probed for CLEC16A and Hrs. The membranes were washed and incubated with a respective mouse/rabbit secondary antibody and bound antibody was detected with WesternBright ECL kit (Advansta). Membranes were stripped and re-probed for β-actin as a loading control.

## IFN-γ ELISA

Human IFN-γ was detected using a DouSet ELISA system (R&D System). YTS and YTS-*CLEC16A* cells (10^6^ cell/ml) where cultured for 0, 30, 120, and 240 min either alone, or activated with 100 ng/ml PMA plus 1 μg/ml ionomycin (PMA+I) (Sigma-Aldrich) or the plate-bound anti-CD28 (5 μg/ml, Abcam) or anti-NKp30 (5 μg/ml, R&D Systems) and saturating amounts of CD226 anti-DNAM-1/CD226 antibody (clone DX11, Low Endotoxin). Supernatants were collected at indicated time points and analyzed by the DouSet ELISA system, following the manufacturer's instructions.

### Mouse Cytokine Array

The Proteome Profiler Mouse Cytokine Array Kit, Panel A (ARY006, R&D Systems) was used to quantify the cytokine/chemokine profile. Briefly, plasma was diluted and mixed with a cocktail of biotinylated detection antibodies. The sample/antibody mixture was then incubated with the array membrane overnight at 4 °C. The membranes were washed and incubated with streptavidin-horseradish peroxidase, followed by chemiluminescent detection. The array data were quantitated to generate a protein profile and results were presented as average signal (pixel density) of the pair of duplicate spots, representing each cytokine or chemokine analyzed using Image-J software.

### NK Cell Cytotoxicity Assay

4-h standard ^51^Cr release assays were performed. Briefly, YTS, YTS-GFP, YTS-*CLEC16A*, and *ex vivo* NK cells were used as effectors and ^51^Cr-labeled K562 erythroleukemia cells, or 721.221 B lymphoblastoid cells were used as targets at effector-to target cell (E:T) ratios of 20:1, 10:1, 5:1, 2.5:1, 0.6:1, and 0.3:1. Mouse splenocytes were used as effectors against the ^51^Cr-labeled YAC-1 murine T-lymphoma cell line at effector-to target cell (E:T) ratios of 50:1, 25:1, 12.5:1, 6.25:1, and 3.12:1. *In vitro* IL-15 stimulation of murine splenocytes was performed by adding 100 ng/ml of rmIL-15 at the start of the cytotoxicity assay where indicated. NK cell cytotoxicity was defined as the lysis of K562, 721.221, or YAC-1 target cells. All test conditions were performed in triplicate, and supernatants harvested after 4 h of incubation at 37°C were evaluated for the presence of ^51^Cr using a Top Count XL counter and Lumiplate scintillation system (Beckman Coulter). Specific lysis was calculated as follows: % Specific lysis = [(mean of the test wells)-(mean of the spontaneous release wells)/(mean of maximal release wells)-(mean of the spontaneous release wells)] × 100%. Maximal release was determined by counts obtained after the incubation of target cells in 0.5% NP40.

### RNA Interference and Nucleofection

*CLEC16A* or control small interfering RNA (siRNA) were introduced via nucleofection into YTS, YTS-*CLEC16A* (program T-20 with reagent R; Amaxa), and *ex vivo* NK cells (program U-O1 with human NK cell nucleofector kit; Amaxa) (Thermo Scientific). Titration experiments were performed to determine the optimal concentration of siRNA. 3 × 10^6^ cells per cuvette, and 24 h of incubation post nucleofection were ultimately selected as optimal conditions. NK cell cytotoxicity was assessed immediately after the 24 h incubation period following nucleofection. Where specified, nucleofected YTS, YTS-*CLEC16A*, and *ex vivo* NK cells whole cell lysates were evaluated by western blot analysis. YTS, YTS-*CLEC16A*, and *ex vivo* NK cells were maintained at 37°C in 5% CO_2_ in complete RPMI medium (Gibco), where “complete” indicates RPMI medium 1640 supplemented with 10% FCS and L-glutamine, minimal essential amino acids, sodium pyruvate, HEPES, and penicillin-streptomycin (Gibco).

### Confocal Microscopy

**Effectors only:** YTS or YTS-*CLEC16A* were adhered to silane coated microscopy slides (Electron Microscopy Sciences) for 20 min, washed twice with DPBS Calcium and Magnesium-free (Life Technologies), and fixed and permeabilized using BD Cytofix/Cytoperm solution (BD Biosciences) for 20 min at room temperature (RT). Cells were washed with BD Perm/Wash buffer supplemented with 2% Bovine Serum Albumin (BSA, Sigma Aldrich). Cells were stained with polyclonal Rabbit-anti-human CLEC16A antibody (Abcam) at a 1:100 dilution for 1 hr. Cells were then washed 3x with Perm/Wash buffer with 2% BSA and probed with secondary polyclonal Goat-anti-Rabbit Alexa Fluor 594 antibody (Abcam) at a 1:200 dilution for 30 min. Cells were washed 3x with Perm/Wash buffer with 2% BSA and stained for F-actin with Phalloidin Alexa Fluor 532 (1:150) (Life Technologies), and monoclonal mouse-anti-human Perforin Alexa Fluor 488 (1:50, BioLegend) for 30 min. Slides were mounted using Corning 22 × 22 mm coverslips No. 1 (Fisher Scientific) and ProLong Gold antifade mountant with DAPI (Life Technologies). All antibody staining was performed at room temperature and antibody dilutions were prepared in Perm/Wash buffer with 2% BSA. **Conjugates**: YTS or YTS-*CLEC16A* effectors were incubated with 721.221 targets in RPMI complete (RPMI, 2 mM L-glutamine, MEM non-essential amino acid solution, 10 mM HEPES, 2 mM sodium pyruvate) (Life Technologies) and 10% heat inactivated Fetal Bovine Serum (Atlanta Biologicals) for a total of 30 min at 37°C. During the first 15 min, effectors and targets were incubated in FACS tubes (BD Biosciences), then briefly vortexed and transferred to silane coated microscope slides for a further 15 min incubation at 37°C. Conjugates were fixed, permeabilized, and stained as described above. **Imaging*:*** All confocal microscopy was performed using the Leica TCS SP8 inverted laser scanning confocal microscope (Leica Microsystems). Images were collected using the Leica HCX PL Apo 100x oil immersion objective (NA = 1.4). DAPI was excited using the 405 nm diode laser, Alexa Fluor 488, 532, and 594 were excited in sequences using a white light laser to avoid cross illumination and bleed through. The Leica LAS AF software was used for image acquisition. Imaging of CLEC16A was performed using the resonant scanner at 12,000 Hz, 512 × 512 pixels, a zoom factor of 5 for detailed images as shown in Figure 2A, and a zoom of 3 for quantitative analyses with a z-step size of 1 μm. Imaging of LC3 was performed under similar conditions with the exception of the imaging mode set to FOV scanner with a frequency of 600 Hz. **Image Analysis**: Quantitative analysis of CLEC16A was performed using Imaris 8.2 image analysis software (Bitplane). Cells were segmented based on the actin staining and isolated using nuclear staining. CLEC16A and perforin vesicles were detected using the spots feature in Imaris. Colocalization analyses were calculated using an ImarisXT plugin: percent of colocalized vesicles was determined by dividing the volume of colocalized vesicles by the total volume of vesicles × 100. Quantitative analyses of LC3 were performed in Volocity 6.3 3D image analysis software (Perkin Elmer). A region of interest was manually drawn around each cell and the threshold for detection of LC3 vesicles was based on intensity and diameter of >0.01 μm. Total CLEC16A or LC3 were determined by multiplying the volume of vesicles with the mean fluorescence intensity per cell. All statistical tests were performed in Prism 7.0 (GraphPad).

### Mice

All animal studies were approved by the Institutional Animal Care and Use Committee of the Children's Hospital of Philadelphia. UBC-*Cre-Clec16a*^loxP/loxp^ mice were generated as described previously (19). All mice were kept on mixed background C57BL/6-129S1. Mice were group-housed on an individually-ventilated cage rack system on a 12:12 light: dark cycle. Mice were fed standard rodent chow and water ad libitum. Ten-week-old mice were treated with tamoxifen (100 mg/kg/day) to induce knockout of *Clec16a* (*Clec16a* KO) or vehicle (10%: ethanol: 90% corn oil) in a control group by gavage at 24-h intervals for 4 consecutive days.

### Statistical Analysis

All graphs denote mean values, and error bars represent the SE. Data were analyzed using unpaired Student's *t*-test as applicable using Prism 7.0 (GraphPad). Differences were considered significant at ^*^P < 0.05, ^**^P < 0.01, and ^***^P < 0.001. The number of mice or pooled samples per experimental group is indicated with “n” in each corresponding figure legend.

## Ethics Statement

All animal studies were approved by the Institutional Animal Care and Use Committee of the Children's Hospital of Philadelphia (IACUC NO: IAC 17-000979). The animals' care was in accordance with the institutional guidelines.

## Author Contributions

RP and MB designed and performed the experiments, analyzed the data, and wrote the manuscript. AY performed confocal microscopy. BS did all animal handling, breeding, and treatment. HSH edited the manuscript. JK and JO provided experimental guidance. HH supervised the entire project and edited the manuscript. All experiments were done in the HH laboratory.

### Conflict of Interest Statement

The authors declare that the research was conducted in the absence of any commercial or financial relationships that could be construed as a potential conflict of interest.
