# Supplementary material for: The Autoimmune Disorder Susceptibility Gene CLEC16A Restrains NK Cell Function in YTS NK Cell Line and Clec16a Knockout Mice
Source: Front Immunol. 2019 Feb 1;10:68. doi: 10.3389/fimmu.2019.00068 (PMC6367972; doi:10.3389/fimmu.2019.00068)
Supplement: Supplementary file 1 [file Presentation_1.pdf]

Supplementary Figure S1

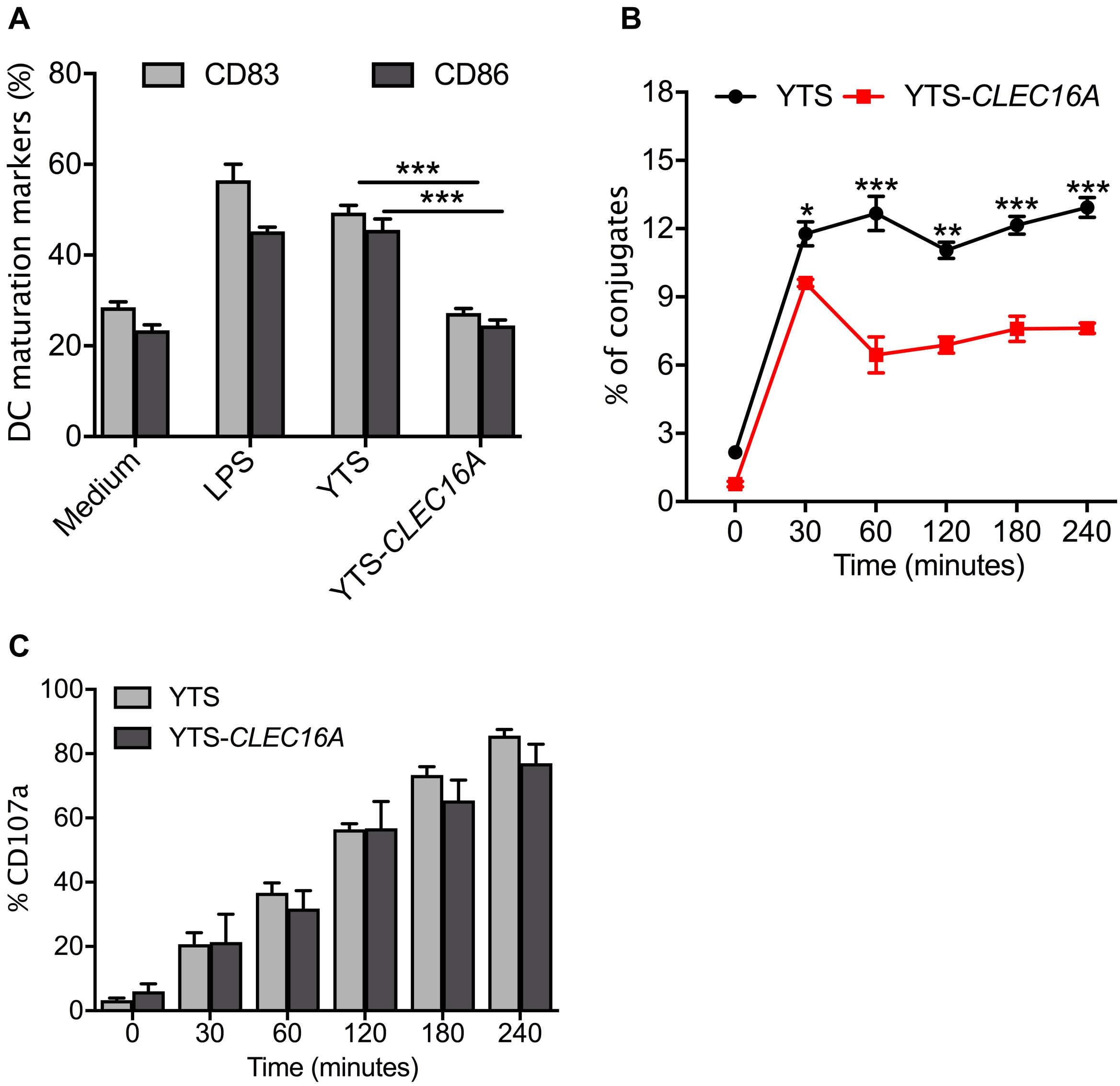

Supplementary Figure S1. (A) CLEC16A overexpression delays NK-dependent DC maturation. Expression of maturation markers on DCs following co-culturing with different YTS and YTS-*CLEC16A* NK cell lines. Controls included immature dendritic cells (iDCs) cultured either in medium alone or in the presence of LPS (1μg/ml). **(B)** Graph depicts kinetics of conjugate formation from 0 min to 240 min period in a fixed cell conjugation assay. **(C)** Percentage of YTS and YTS-*CLEC16A* conjugated NK cells expressing CD107a following stimulation with 721.221 targets in a time course experiment. Data represents means±SE of three independent experiments (n=3). **P*<0.05, ***P*<0.01, ****P*<0.001 (unpaired two-tailed Student's t-test).

Supplementary Figure S2

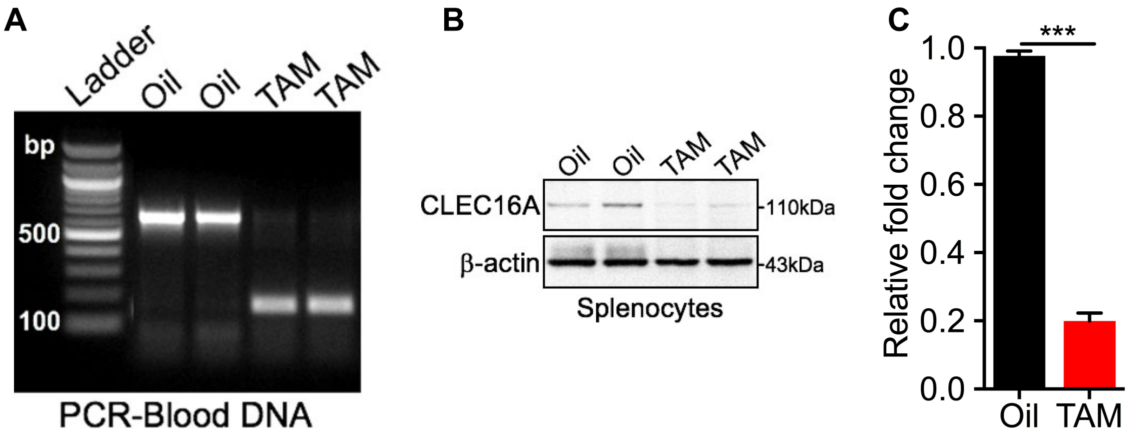

Supplementary Figure S2. (A) PCR analysis of genomic DNA isolated from whole blood of KO (TAM) and controls (oil) mice show PCR products of 618bp in controls and 146bp in KO, confirming a removal of *Clec16a* exon 3 in KO mice. **(B)** Representative Western blot of CLEC16A expression in murine splenocytes. **(C)** Quantitation graph depicting CLEC16A protein expression (n=3 repeats). ****P*<0.001 (unpaired two-tailed Student's t-test).

Supplementary Figure S3

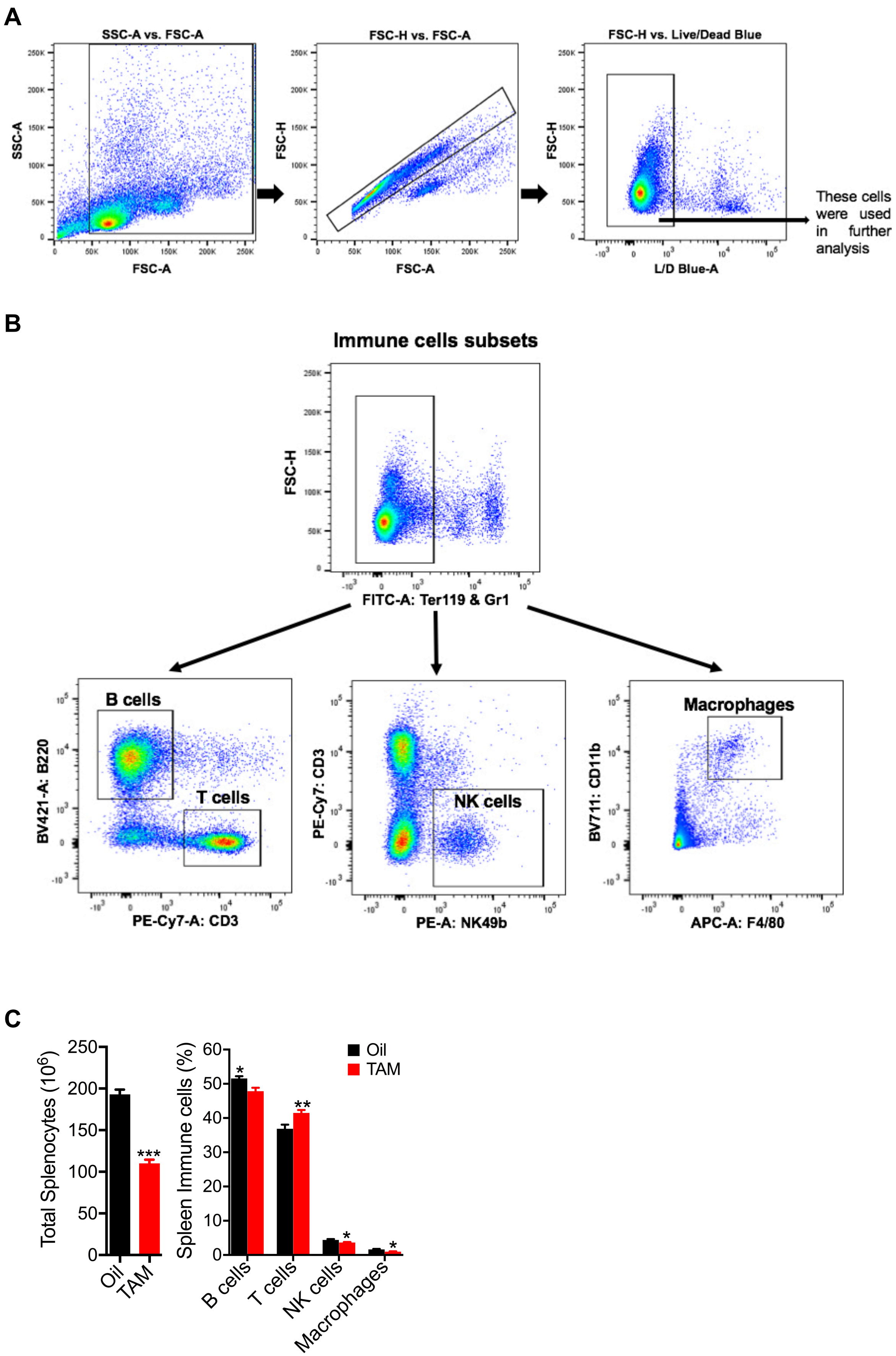

Supplementary Figure S3. (A) Total mouse splenocytes were prepared as described in Methods. Debris (SSC-A vs. FSC-A) and doublets (FSC-H vs. FSC-A) were excluded and live/dead discrimination was determined using the Live/Dead Blue dye (FSC-H vs. Live/Dead Blue). **(B)** Gating strategy for identification of different host immune cell subsets present in mouse spleen is shown. As described previously debris, doublets and nonviable cells were excluded from total mouse splenocytes. Ter-119^{+} and Gr-1^{+} cells were omitted from subsequent analysis B220^{+} cells were defined as B cells. CD3^{+} were defined as T cells. F4/80^{+} Mac-1^{+} cells were defined as monocytes/macrophages. NK1.1^{+}CD3^{-} were defined as NK cells. **(C)** Reduced spleen cell numbers and altered splenic Immune cell population in *Clec16a* KO (TAM) mice (n=12 mice per group). Data represents means±SE of three independent experiments (n=3). **P*<0.05, ***P*<0.01, ****P*<0.001 (unpaired two-tailed Student's t-test).

Supplementary Figure S4

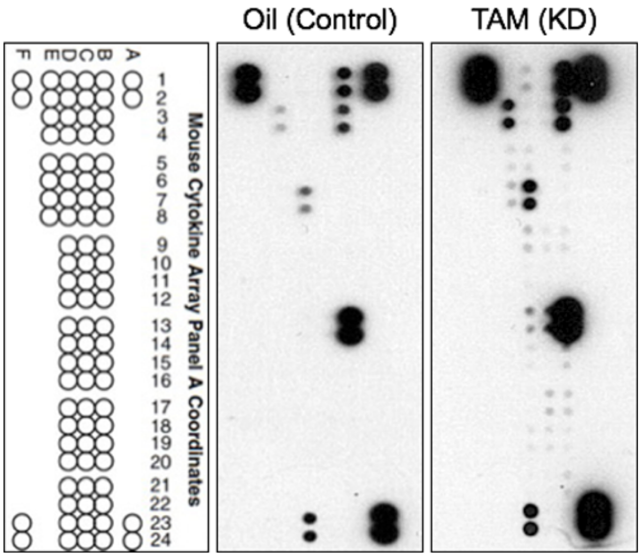

Supplementary Figure 4. Mouse Cytokine Array. (**A**) Representative Array blot of the plasma cytokine and chemokine from Control (Oil), and KD (TAM) mice. 100 ul of plasma was run on the array. Image shown is from a two-hour exposure to X-ray film. The average signal (pixel density) of the pair of duplicate spots representing each cytokine or chemokine was analyzed using Image-J software (**B**). Table depicts cytokines, chemokines, adipokines, growth factors and immune related proteins coordinates on Mouse Cytokine Array Panel A.

Coordinate	Target/Control
A1, A2	Reference Spot
A23, A24	Reference Spot
B1, B2	BLC (CXCL13/BCA-1)
B3, B4	C5/C5a
B5, B6	G-CSF
B7, B8	GM-CSF
B9, B10	I-309 (CCL1/TCA-3)
B11, B12	Eotaxin (CCL11)
B13, B14	sICAM-1 (CD54)
B15, B16	IFN-γ
B17, B18	IL-1α
B19, B20	IL-1β
B21, B22	IL-1ra
B23, B24	IL-2
C1, C2	IL-3
C3, C4	IL-4
C5, C6	IL-5
C7, C8	IL-6
C9, C10	IL-7
C11, C12	IL-10
C13, C14	IL-13
C15, C16	IL-12p70
C17, C18	IL-16
C19, C20	IL-17
C21, C22	IL-23
C23, C24	IL-27
D1, D2	IP-10 (CXCL10/CRG-2)
D3, D4	I-TAC(CXCL11)
D5, D6	KC (CXCL1)
D7, D8	M-CSF
D9, D10	JE (CCL2/MCP-1)
D11, D12	MCP-5(CCL12)
D13, D14	MIG (CXCL9)
D15, D16	MIP-1α (CCL3)
D17, D18	MIP-1β (CCL4)
D19, D20	MIP-2 (CXCL2)
D21, D22	RANTES (CCL5)
D23, D24	SDF-1 (CXCL12)
E1, E2	TARC
E3, E4	TIMP-1
E5, E6	TNFα
E7, E8	TREM-1
F1, F2	Reference Spot
F23, F24	PBS (negative Control)

Supplementary Figure S5

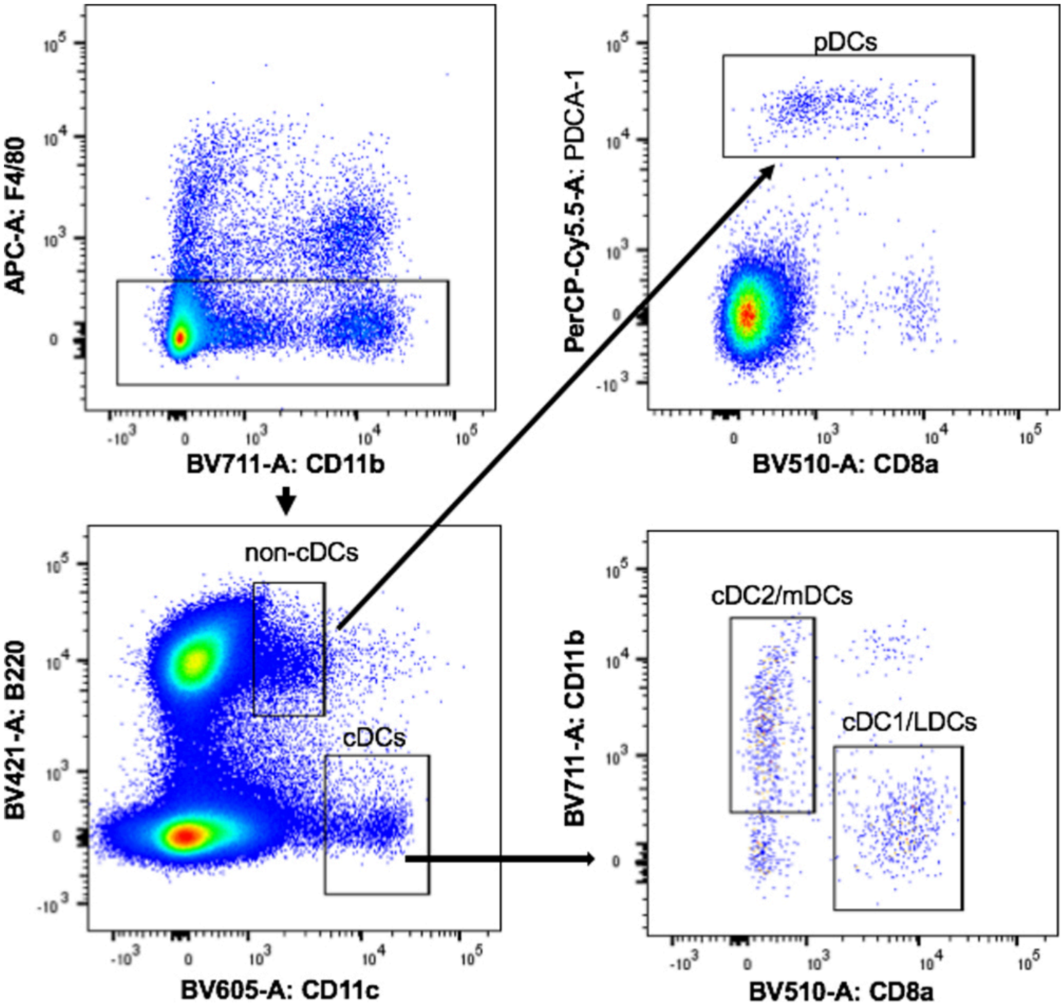

Supplementary Figure S5. Immunophenotyping of dendritic cell subsets in mouse spleens. A gating strategy for identification of different host dendritic cell subsets present in mouse spleens is shown. As described previously debris, doublets and non-viable cells were excluded from total mouse splenocytes. Dot plot of F4/80 against CD11b was used for gating out splenic macrophages ($F4/80^{+}$). Since the selected fraction contained both conventional dendritic cells (cDCs) and non-cDCs, the two populations were separated from each other by plotting B220/CD45R against CD11c, which helped in differentiating between cDCs ($CD11c^{hi}B220/CD45R^{-}$) and non-cDCs ($CD11c^{low}B220/CD45R^{+}$). The cDCs were further subgated into either cDC2/myeloid DC (mDCs) or cDC1/lymphoid DC (LDCs) based on the expression of CD11b and CD8a surface markers: while cDC2 were $CD11c^{+}CD11b^{+}CD8^{-}$, cDC1 were $CD11c^{+}CD11b^{-}CD8^{+}$. Similarly, from the non-cDc pool, a dot plot of PDCA-1 against CD8a yielded plasmacytoid DCs (pDCs) $B220/CD45R^{+}CD11c^{low}PDCA1^{+}$.

Supplementary Figure S6

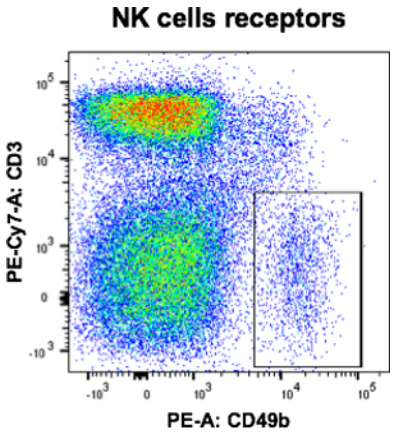

Supplementary Figure S6A. A gating strategy for identification of natural killer (NK) cells present in mouse spleen is shown. As described previously debris, doublets and nonviable cells were excluded from total mouse splenocytes. Thereafter, cell populations within the PE-Cy7-A (CD3) versus PE-A (CD49b) dot plot were segregated according to their differential profiles of the above-mentioned markers. CD3-CD49b+ NK cells were easily identified. Further analysis for NK cell receptors expression was performed when gated on the sub-population of NK cells. Each sample was split in 7 sets to asses NK receptors.

Set 1: BV421-CD122, FITC-Ly49D, AF647-2B4
Set 2: FITC-Ly49A, AF647-Ly49H, BV711-NKp46
Set 3: FITC-CD94, AF647-CD226, BV711-NKG2D
Set 4: FITC-Ly49C/I, AF647-Qa2
Set 5: AF700-CD69
Set 6: BV421-CD44, FITC-CD2, APC-CD43
Set 7: BV421-CD11b, FITC-CD11a, APC-CD28

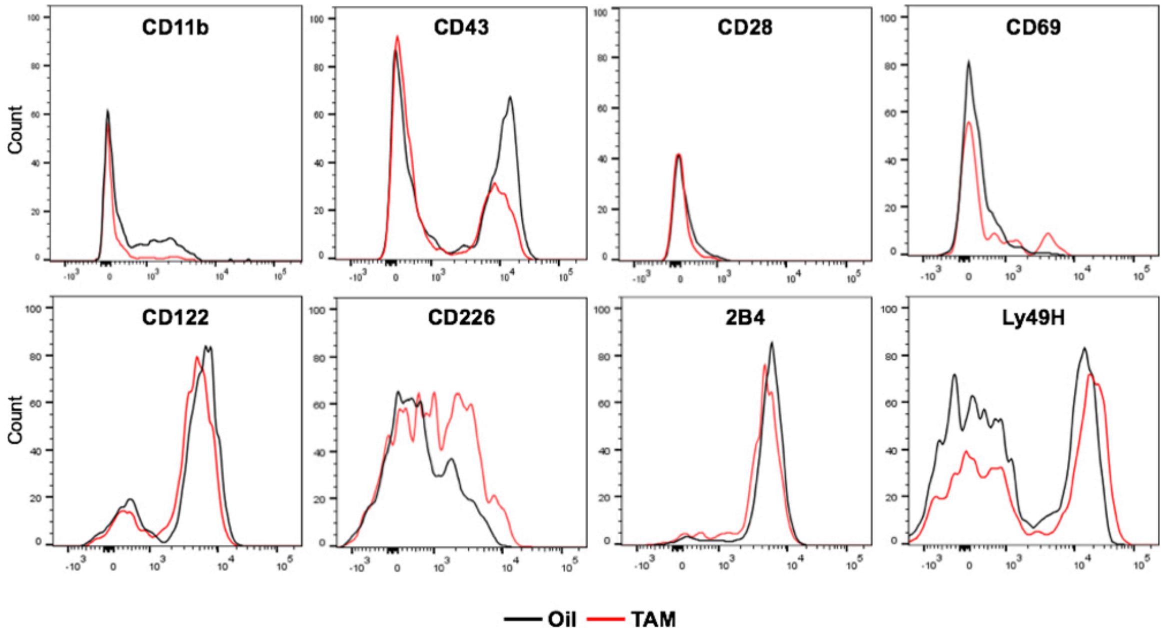

Supplementary Figure S6B. Representative histogram depicting expression of CD11b, CD43, CD28, CD69, CD122, CD226, 2B4 and Ly49H on NK cells in control and knockout mice.

Supplementary Table 1. Antibodies used in Flow assays	Resource	Identifier
1. PE anti-human CD337 (NKp30)	BioLegend	325208
2. PE anti-human CD28	BioLegend	302908
3. PE anti-human CD335 (NKp46)	BioLegend	331908
4. PE anti-human CD226 (DNAM-1)	BioLegend	338306
5. PE anti-human CD314 (NKG2D)	BioLegend	320806
6. PE anti-human CD11a	BioLegend	301208
7. PE anti-human CD16	BioLegend	302008
8. PE anti-human CD18 Antibody	BioLegend	302107
9. Alexa Fluor® 647 anti-human CD107a (LAMP-1)	BioLegend	328612
10. PE anti-human CD83	BioLegend	305307
11. APC anti-human CD86	BioLegend	305411
12. PerCP/Cy5.5 anti-human CD1a	BioLegend	300129
13. FITC anti-human CD14	BioLegend	325603
14. PE anti-mouse CD49b	BioLegend	103506
15. PE/Cy7 anti-mouse CD3ε	BioLegend	100320
16. Brilliant Violet 421™ anti-mouse/human CD45R/B220	BioLegend	103240
17. APC anti-mouse F4/80	BioLegend	123116
18. FITC anti-mouse Ly-6G/Ly-6C (Gr-1)	BioLegend	108406
19. FITC anti-mouse TER-119/Erythroid Cells	BioLegend	118206
20. Brilliant Violet 711™ anti-mouse/human CD11b	BioLegend	101242
21. FITC anti-mouse CD94	BioLegend	105506
22. FITC anti-mouse Ly-49A	BioLegend	116805
23. Alexa Fluor® 647 anti-mouse Qa-2	BioLegend	121708
24. Alexa Fluor® 647 anti-mouse CD244.2 (2B4 B6 Alloantigen)	BioLegend	133510
25. Alexa Fluor® 647 anti-mouse CD226 (DNAM-1)	BioLegend	133606
27. Brilliant Violet 711™ anti-mouse CD335 (NKp46)	BioLegend	137621
28. FITC anti-mouse Ly49D	BioLegend	138303
29. Alexa Fluor® 647 anti-mouse Ly49H	BioLegend	144710
30. Alexa Fluor® 700 Hamster Anti-Mouse CD69	BD Pharmingen	561238
31. FITC Mouse Anti-Mouse Ly-49C and Ly-49I	BD Pharmingen	562055
32. Brilliant Violet 421™ Rat Anti-Mouse CD122	BD Pharmingen	562960
33. Brilliant Violet 711™ Rat Anti-Mouse CD314 (NKG2D)	BD Pharmingen	563694
34. Brilliant Violet 421™ anti-mouse/human CD44	BioLegend	103039
35. FITC anti-mouse CD2	BioLegend	100105
36. APC anti-mouse CD43	BioLegend	143208
37. Brilliant Violet 421™ anti-mouse/human CD11b	BioLegend	101235
38. FITC anti-mouse CD11a	BioLegend	101106
39. APC anti-mouse CD28	BioLegend	102109
40. Brilliant Violet 510™ anti-mouse CD8a	BioLegend	100751
41. Brilliant Violet 605™ anti-mouse CD11c	BioLegend	117333
42. Alexa Fluor® 488 anti-mouse I-A/I-E	BioLegend	107615
43. PE anti-mouse CD80	BioLegend	104707
44. PerCP/Cyanine5.5 anti-mouse CD317 (BST2, PDCA-1)	BioLegend	127021
45. APC/Cy7 anti-mouse CD86	BioLegend	105029
